# Effects of Different PER Translational Kinetics on the Dynamics of a Core Circadian Clock Model

**DOI:** 10.1371/journal.pone.0115067

**Published:** 2015-01-21

**Authors:** Paula S. Nieto, Jorge A. Revelli, Eduardo Garbarino-Pico, Carlos A. Condat, Mario E. Guido, Francisco A. Tamarit

**Affiliations:** 1 Instituto de Física Enrique Gaviola (IFEG-CONICET) and Facultad de Matemática, Astronomía y Física (FaMAF), Universidad Nacional de Córdoba (UNC). Ciudad Universitaria, CP:X5000HUA Córdoba, Argentina; 2 Centro de Investigaciones en Química Biológica de Córdoba (CIQUIBIC-CONICET) and Departamento de Química Biológica, Facultad de Ciencias Químicas, Universidad Nacional de Córdoba (UNC). Ciudad Universitaria, CP:X5000HUA Córdoba, Argentina; Karlsruhe Institute of Technology, GERMANY

## Abstract

Living beings display self-sustained daily rhythms in multiple biological processes, which persist in the absence of external cues since they are generated by endogenous circadian clocks. The *period* (*per*) gene is a central player within the core molecular mechanism for keeping circadian time in most animals. Recently, the modulation PER translation has been reported, both in mammals and flies, suggesting that translational regulation of clock components is important for the proper clock gene expression and molecular clock performance. Because translational regulation ultimately implies changes in the kinetics of translation and, therefore, in the circadian clock dynamics, we sought to study how and to what extent the molecular clock dynamics is affected by the kinetics of PER translation. With this objective, we used a minimal mathematical model of the molecular circadian clock to qualitatively characterize the dynamical changes derived from kinetically different PER translational mechanisms. We found that the emergence of self-sustained oscillations with characteristic period, amplitude, and phase lag (time delays) between *per* mRNA and protein expression depends on the kinetic parameters related to PER translation. Interestingly, under certain conditions, a PER translation mechanism with saturable kinetics introduces longer time delays than a mechanism ruled by a first-order kinetics. In addition, the kinetic laws of PER translation significantly changed the sensitivity of our model to parameters related to the synthesis and degradation of *per* mRNA and PER degradation. Lastly, we found a set of parameters, with realistic values, for which our model reproduces some experimental results reported recently for *Drosophila melanogaster* and we present some predictions derived from our analysis.

## Introduction

Predictable environmental changes such as the daily variation in ambient lighting, temperature, nutrients, etc., have been critical for the temporal organization of most living beings. Organisms have developed *endogenous* circadian clocks, which generate most of the daily variations in their molecular biology, biochemistry, physiology and behavior with a period ∼ 24 *h*. These circadian rhythms are self-sustained and persist in the absence of external clues. Importantly, circadian clocks are entrained by periodic environmental cues and allow organisms to anticipate their physiology to these external changes [1].

Nearly all metazoans studied so far have at least one circadian oscillator within each cell, which is thought to enhance cellular fitness by temporally regulating key metabolic and physiological processes. At the single cell level, the circadian clockwork is based on a set of genes, called *clock genes*, whose protein products (clock proteins) are necessary components for the generation and regulation of circadian rhythms. Most clock genes are expressed in a circadian fashion as a consequence of their mutual interaction and regulation. The mechanism by which clock genes and proteins interact with one another is commonly called the *molecular clock mechanism* and is based on interconnected transcriptional-translational feed back loops (TTFL). By means of this mechanism, the clock genes also regulate downstream specific output genes (called clock-controlled genes, *ccgs*) in a circadian manner [2]. The *period* (*per*) gene is a central player within the core molecular mechanism for keeping circadian time in most animals. However, other clock components are also required to sustain the *per* feedback loop [3].

The complexity of the molecular circadian rhythmicity has been largely unraveled through experimental studies but also by the use of mathematical models, which are powerful tools able to yield and explain non-intuitive results [2, 4, 5]. Goldbeter [6] developed the first mathematical model for the *Drosophila* molecular circadian clock based on the negative feedback exerted by the PER protein over its own transcription. Similar kinetic models have subsequently been used by many authors to describe diverse aspects of molecular circadian rhythmicity such as the entrainment by light-dark cycles, responses to specific drugs, etc [5, 7–14]. Moreover, stochastic versions of these models were also developed in order to study the robustness of molecular rhythmicity with respect to molecular noise [9, 15, 16].

Experimental and theoretical studies have always considered transcription as the main mechanism by which it is possible to exert temporal control over gene expression [17, 18]. However, in recent years an increasing number of reports have stressed the importance of post-transcriptional mechanisms as an alternative level of gene expression regulation [19, 20]. Specifically, translational modulation is now recognized as a major post-transcriptional mechanism for the control of gene expression in a wide range of cellular conditions. This process allows a rapid fine tuning of protein levels, in both time and space, without requiring transcription of new mRNAs or post-translational regulation [21–24]. In addition, translational regulation of some clock components, like PER, have been recently described in flies and mammals. For instance, both, the protein TWENTY FOUR (TYF) and the non-canonical translation factor NAT1 enhance PER translation in flies [25, 26]; whereas in mammals, two different RNA-binding proteins, LARK and hnRNP-Q, bind independently to *per1* mRNA, enhancing its translation [27, 28]. It is important to note that although the modulation of PER translation has been observed experimentally, this post-transcriptional regulatory process was never incorporated into mathematical circadian clock models. Furthermore, theoretical studies of the molecular circadian clock have never analyzed in detail how the translational kinetics affects the oscillatory properties of the circadian molecular clock.

Here we hypothesize that the *effective* kinetics of PER translation is the result of the fine modulation of PER synthesis by translational regulators (*R*). Accordingly, we posit that the regulation of PER translation involves changes in its translational kinetics, which ultimately affects the molecular clock dynamics. Therefore, the main goal of this work is to characterize how kinetically different mechanisms of PER translation affect the dynamical behavior of the molecular clock.

By using a single-cell model for the circadian molecular clock [6, 9, 29], we performed a systematic and novel study of how the oscillatory properties (period, amplitude and time delays -i.e.: time lags between the *per* mRNA and protein expression-) of a core circadian clock model are affected by the kinetics of PER translation. We propose a *master* clock model from which we can study different particular cases by specifying the values of translation-related parameters. This *master* clock model includes two kinetically different mechanisms of PER translation, one with a first order kinetics and the other with a Michaelis-Menten kinetics. Through the introduction of a Michaelis-Menten term, we have performed a deep characterization of whether, and to what extent, the introduction of nonlinearities in the translational kinetics affects the dynamical properties of the system, either when the translation occurs by a single kinetic mechanism (characterized only by the parameters of the Michaelis-Menten term) or by the sum of two kinetically different mechanisms. We have interpreted our model according to experimental observations and the results obtained as a consequence of the regulation of PER synthesis. We found that the emergence of self-sustained oscillations, as well as the period, amplitude, and time delays values depend on the kinetic parameters related to PER translation. Interestingly, under certain conditions, a PER translation mechanism with saturable kinetics introduces longer time delays than a mechanism ruled by a first-order kinetics. In addition, we found that the kinetic laws of PER translation significantly changed the sensitivity of our model with regard to parameters related to the *per* mRNA synthesis and PER degradation. Lastly, we found a set of parameters, with realistic values, for which our model reproduces some experimental results reported recently for *Drosophila melanogaster* [25, 26], and we present some predictions derived from this study.

## The molecular circadian clock model

In this work we will mainly refer to the *Drosophila melanogaster* molecular clock, but the same principles (i.e.: TTFL mechanism) are shared with the mammalian molecular clock [2, 30]. In the *Drosophila* core feedback loop, the clock proteins CLOCK (CLK) and CYCLE (CYC) heterodimerize and bind to regulatory sequences (E-boxes) present in the promoter of target clock genes such as *per*, *timeless* (*tim*) and *doubletime* (*dbt*). These interactions promote the transcriptional activation of such clock genes, yielding their corresponding mRNAs. Once they are translated, the PER and TIM proteins start accumulating in the cytoplasm, about 6—8 *h* after their respective mRNAs. The DOUBLETIME (DBT) protein binds to PER and phosphorylates it, whereas TIM stabilizes the DBT-PER complexes, enabling the accumulation of DBT-PER-TIM complexes in the cytoplasm. Nuclear localization of such complexes is boosted by the phosphorylation of PER and TIM by CASEIN KINASE 2 (CK2) and SHAGGY (SGG), respectively. Within the nucleus, PER-TIM (and /or DBP-PER-TIM) complexes bind to CLK, promoting the release of the heterodimer CLK-CYC from the E-boxes. PER-TIM complexes therefore repress their own transcription by inhibiting the transcriptional activity of CLK-CYC. When PER levels decay because of degradation, CLK accumulates, the CLK-CYC heterodimer can bind to the E-boxes and a new loop begins [3]. Additional feedback loops such as the *clk* and *cwo* loops, are interlocked with the core loop, conferring robustness to the mechanism and supporting circadian rhythmicity in flies. Furthermore, kinases, phosphatases and other enzymes post-translationally modify clock proteins, which also modulate molecular circadian rhythmicity [3].

This conceptual model of how the *Drosophila* molecular clock is organized and works can be transformed into a mathematical model by the selection of suitable mathematical equations and parameter values in order to represent the biochemical processes involved [31]. Indeed, numerous mathematical models, based on the Goodwin oscillator, have been developed by many authors [5, 7–14, 32]. The complexity of these deterministic models has evolved according to the identification of new clock components and processes, which have generated very detailed models that attempt to mimic the exact behavior of the molecular clock [31, 32]. The main drawback of those very detailed models is the large number of parameters involved, many of which have experimentally unknown values [31, 32]. As discussed previously [33], a very detailed mathematical model does not guarantee good consistency between the results of the model and experimental data. On the other hand, less detailed mathematical models, such as the Goldbeter model [6] and its derivatives [9, 12, 14, 29], include core processes involved in the negative feedback exerted by a regulatory protein (PER) on the expression of its own gene, keeping the model as simple as possible. Indeed, in some cases, these less detailed mathematical models produced results more similar to what it is observed experimentally [33], and are more suitable for studying the underlying properties of the feedback loop systems [31, 32]. This is the focus of our work and, therefore, we based our study on a Goldbeter structure-based model because, to our judgement, this model describes quite rigorously the *per* mRNA and protein dynamics, with (relatively) few parameters [6].

**Figure 1:**
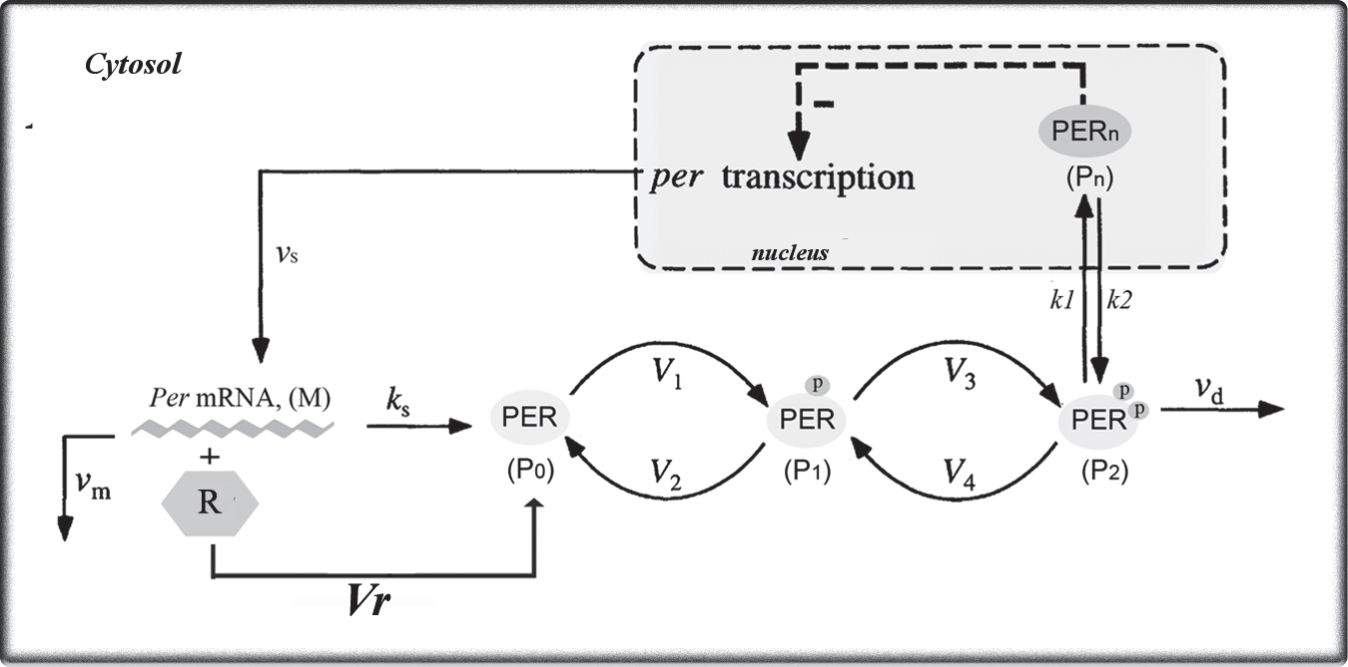
Scheme of the ‘master’ model proposed in the present work. The model is based on a Goodwin oscillator in which the temporal variation of the concentrations of mRNA (*M*) and the various forms of clock protein, cytosolic (*P_0_*, *P_1_*, *P_2_*) or nuclear (*P_n_*), are governed by the system of kinetics equations 1–5. The associated parameters with their specific values are indicated in S1 Table in S1 File. This scheme is similar to the one presented in [6].

We took as a starting point of our work the deterministic model for the *Drosophila* molecular clock proposed by Gonze and co-workers [9]. Gonze’s deterministic version has exactly the same functional form (terms and equations) as the original model published by Goldbeter in 1995 [6]. The differences between these two models are exclusively related to the parameters values, which we assumed that were improved in the more recent paper [9]. In our model (Fig. 1), most of the core processes involved in circadian rhythmicity were assumed to occur with kinetics identical to those described in previous reports [9, 29], except for PER translation. Thus, our model relies on the following assumptions discussed below.

*per* mRNA (*M*) is transcribed into the nucleus and transferred to the cytoplasm at a rate *v_s_* (*nM h* ^−*1*^), where it decays in a Michaelis-Menten fashion with rate *v_m_* (*nM h* ^−*1*^) and Michaelis constant *K*_m_ (*nM*). In the cytoplasm, the *per* mRNA can be translated to produce the PER protein. The exact functional form of the PER translation is not known experimentally, except that it is modulated by some regulators that enhance the PER translation [25, 26]. Besides, since multiple regulators and factors are involved in the effective kinetics of protein synthesis, it is likely that this process occurs with nonlinear kinetics [34, 35]. Therefore, we have made the plausible assumption that the PER translation follows a Michaelis-Menten kinetics. We have chosen a Michaelis-Menten kinetics because it is one of the simplest ways to represent a biochemical-enzymatic reaction with nonlinear kinetics. Thus, by taking into account a Michaelis-Menten kinetics for the PER translation we have incorporated two new parameters to the model: the Michaelis constant *K_r_* (*nM*) and the maximum rate of the translational process *V_r_* (*nM h* ^−*1*^). In classic enzymology the maximum rate *V_max_* is proportional to the concentration of the enzyme involved in the enzymatic reaction. Analogously, here the *V_r_* parameter is proportional to the concentration of multiple translational regulators involved in PER translation, which we generically represent as *R* and are assumed constant across time. Therefore, the PER translation mechanism following a Michaelis-Menten kinetics is dependent on the *R* activity and for simplicity we named it as the *R*-dependent translation. In previous models the PER translation rate was assumed as proportional to the *per* mRNA concentration and characterized by the first-order rate constant *k_s_* (*h* ^−*1*^) [6, 9, 29]. In our model we have maintained this translational mechanism, independent of the *R* activity, referred here as the *R*-independent translation.

Thus, our model takes into account two different mechanisms by which *per* mRNA can be translated: an *R*-independent (characterized by the first-order rate constant *k_s_*) and an *R*-dependent (with Michaelis-Menten kinetics) translation. We have added the *R*-dependent translation in our model, instead of replacing the *R*-independent step, mainly because considering the double mechanism allows us to obtain a *master* model from which we can study different particular cases by specifying the values of *k_s_*, *V_r_* and *K_r_*:

1. by setting *k_s_* = 0, the model considers that the *R*-dependent mechanism is the only one involved in PER translation;
2. by setting *V_r_* = 0, the model takes into account that the *R*-independent translation is the only mechanism involved in PER synthesis. Specifically, when *k_s_* = 2 the model is exactly the same as the original model [9].
3. by setting *K_r_* ≠ 0, *V_r_* ≠ 0 and *k_s_* ≠ 0 we explored how the clock dynamic is affected when both translation mechanisms coexist, but have different weights within the system. We have analyzed the system with a double mechanism of PER translation because several experimental studies have recently revealed that some clock proteins (i.e., dPER in *Drosophila* and PER1 in mammals) could be translated by both cap-dependent and cap-independent mechanisms, although the relative contribution of such mechanisms to the overall translational process under normal conditions is still unknown experimentally [26, 36].

The proposed mathematical model also takes into account the PER post-translational modifications described in the literature [6, 9, 29]: PER (*P_0_*) is phosphorylated twice in a consecutive and reversible fashion, resulting in the mono-phosphorylated PER (*P_1_*) and the bi-phosphorylated PER (*P_2_*). The first phosphorylation/dephosphorylation step is modeled by a Michaelis-Menten mechanism with a maximum rate *V_1_* (*nM h* ^−*1*^) and a Michaelis constant *K_1_* (*nM*) for the forward reaction and with a maximum rate *V_2_* (*nM h* ^−*1*^) and a Michaelis constant *K_2_* (*nM*) for the reverse reaction. The same kinetics is assumed for the second phosphorylation/dephosphorylation step, with maximum rates *V_3_* (*nM h* ^−*1*^) and *V_4_* (*nM h* ^−*1*^), and Michaelis constants *K_3_* (*nM h* ^−*1*^) and *K_4_* (*nM h* ^−*1*^), respectively. Only biphosphorylated PER (*P_2_*) can be degraded with a Michaelis-Menten kinetics with maximum rate *v_d_* (*nM h* ^−*1*^)and Michaelis constant *K_d_* (*nM*). Nuclear translocation of the bi-phosphorylated PER is considered as a reversible step with first-order rate constants *k_1_* (*h* ^−*1*^) and *k_2_* (*h* ^−*1*^) for the forward and reverse processes, respectively. To simplify the model it was assumed that nuclear PER (*P_n_*) itself is the repressor exerting the negative feedback on *per* expression. The feedback term is described by a Hill-like equation with Hill coefficient *nH*. The bigger the *nH* value, the more cooperative is the repression of *per* transcription.

In his pioneering work, Goldbeter had assumed allosteric kinetics for the repression of *per* transcription, with *nH* = 4 [6], but he also showed that non-cooperative kinetics, with *nH* = 1, can give rise to self-sustained oscillations. Forger and coworkers [11] suggested that there are non-allosteric interactions among E-boxes, and proposed a non-cooperative *per* transcription. Recently, Brown and collaborators have supported the idea that repression must be a cooperative process [37]. In this work we have therefore taken into account both cooperative and non-cooperative *per* repression mechanisms and we compared the results obtained in each case.

The total protein (*P_t_*) is calculated as the sum of all PER species (*P_t_* = *P_0_* + *P_1_* + *P_2_* + *P_n_*).

The differential equations of this model are:

$$\frac{dM}{dt}\;=v_{s}\frac{K_{i}^{nH}}{K_{i}^{nH}+P_{n}^{nH}}-v_{m}\frac{M}{K_{m}+M}$$ (1)

$$\frac{dP_{0}}{dt}\;=k_{s}M+V_{r}\frac{M}{K_{r}+M}-V_{1}\frac{P_{0}}{K_{1}+P_{0}}+V_{2}\frac{P_{1}}{K_{2}+P_{1}}$$ (2)

$$\frac{dP_{1}}{dt}=V_{1}\frac{P_{0}}{K_{1}+P_{0}}-V_{2}\frac{P_{1}}{K_{2}+P_{1}}-V_{3}\frac{P_{1}}{K_{3}+P_{1}}+V_{4}\frac{P_{2}}{K_{4}+P_{2}}$$ (3)

$$\frac{dP_{2}}{dt}=V_{3}\frac{P_{1}}{K_{3}+P_{1}}-V_{4}\frac{P_{2}}{K_{4}+P_{2}}-k_{1}P_{2}+k_{2}P_{n}-v_{d}\frac{P_{2}}{K_{d}+P_{2}}$$ (4)

$$\frac{dP_{n}}{dt}=k_{1}P_{2}-k_{2}P_{n}$$ (5)

It is necessary to clarify here that the nonlinear terms appearing in the kinetic equations do not correspond to single reaction steps. Instead, these terms represent compact kinetic expressions obtained after application of quasi-steady-state hypotheses on enzyme substrate or gene-repressor complexes.

Summarizing, the model consists of a set of five ordinary differential equations describing the time evolution of five components of the molecular circadian clock: *M*, *P_0_*, *P_1_*, *P_2_* and *P_n_*. The system requires 20 parameters, whose values and definitions are listed in S1 Table in S1 File. Unless indicated otherwise, the value for most of the parameters are those listed in S1 Table in S1 File, which are the same than those reported in [9].

## Results

### The oscillatory behavior of the system

We used the deterministic version of the model proposed by Gonze and coworkers [9] as the starting point for our study. First, we characterized the effect of the *R*-dependent translation on the system dynamics, using similar conditions (i.e.: *k_s_* = 2 *h* ^−*1*^) to those previously reported [9, 29]. In order to do this, we studied the dynamical behavior of the system when the *R*-dependent translation has a small contribution to the overall PER translation, that is, when the *V_r_* and *K_r_* parameters are very small compared to the *k_s_* value (i.e.: *k_s_* = 2 *h* ^−*1*^, *K_r_* = 10^−*2*^ *nM* and *V_r_* = 10^−*2*^ *nM h* ^−*1*^).

**Figure 2:**
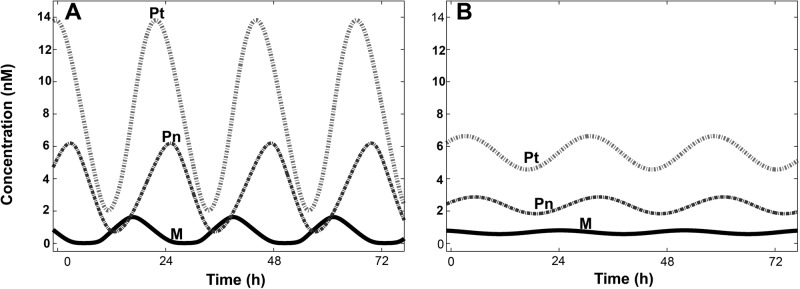
Sustained oscillations generated by the PER model. Temporal evolution of the variables *M*, *P_n_*, and *P_t_*, which represent the *Per* mRNA, nuclear PER and total PER, respectively. Repression of *per* transcription was assumed as a cooperative (*A*) or a non-cooperative (*B*) process. Parameter values are those listed in S1 Table in S1 File, except for the translation-related parameters, which, for this figure, acquired the following values: *k_s_* = 2 *h* ^−*1*^, *K_r_* = 10^−*2*^ *nM* and *V_r_* = 10^−*2*^ *nM h* ^−*1*^.

Fig. 2, *A* and *B*, shows the time evolution of *M*, *P_n_* and *P_t_* for *nH* = 4 and *nH* = 1, respectively. The system displayed sustained circadian oscillations in all the variables, regardless of the assumption of a cooperative (*n* = 4) or a non-cooperative (*n* = 1) repression of *per* transcription. The amplitudes of such oscillations were dramatically lower in the non-cooperative model and power spectrum analysis (S1 Fig. *A* and *B* in S1 File) revealed major differences in the oscillation period (*τ* between the cooperative *τ* ≈ 22.3 *h*) and the non-cooperative (*τ* ≈ 27.7 *h*) cases. S1 Fig. *C* and *D* in S1 File shows a parametric plot of the concentrations of *M* vs *P_t_*, revealing a limit cycle trajectory, which was obtained regardless of the initial conditions for both cooperative and non-cooperative models. These results indicate that for a small contribution of the *R*-dependent translation to the overall translation, the system ruled by Equations 1 to 5 reaches a regime of self-sustained oscillations, regardless of whether the model assumes cooperativity or not. Thus, assuming a cooperative repression of *per* transcription favors the periodic behavior of the system, increasing the amplitude of the oscillations but shortening the period length, in agreement with the results of previous reports [6]. For the cooperative case (*nH* = 4), we have not found major differences in the period (*τ* ≈ 22.3 *h*) or the amplitude (*a*) of the oscillations between the model under the conditions presented in Fig. 2 *A* (*k_s_* = 2 *h* ^−*1*^, *K_r_* = 10^−*2*^ *nM* and *V_r_* = 10^−*2*^ *nM h* ^−*1*^) and the model under the conditions corresponding to the original model (i.e.: *k_s_* = 2 *h* ^−*1*^, *K_r_* = 0 *nM* and *V_r_* = 0 *nM h* ^−*1*^; [9], S2 Fig. *A* in S1 File). For the non-cooperative case (*nH* = 1), on the other hand, we found that the oscillation amplitude significantly decreased when the *R*-dependent translation was considered (S2 Fig. *B* in S1 File) while the period is practically unaffected (*τ* ≈ 27.7 *h*). Together, these results revealed that a small contribution of the *R*-dependent translation to the overall PER translation produced minor changes in the period of the molecular clock as compared to those obtained with the original model [9]. These conclusions are further supported by the evidence emerging from the comparison between the mRNA concentration ([*M*]) and the *K_r_* value (0.01 *nM*). Fig. 2 *A* shows that the [*M*] value at the peak is much bigger than the *K_r_* value ([*M*] ≈ 1.6 *nM* >> *K_r_*) and therefore the Michaelian term can be reduced to a constant term, approximately equal to *V_r_*. The effective PER translational kinetics (that is, the sum of the *R*-dependent and the *R*-independent terms) can be written as (*k_s_* + *Z*)[*M*], with $Z=\frac{V_{r}}{\left[M\right]}$ << *k_s_*, which leads to an effective translational kinetics very close to that considered in the original paper [9]. On the other hand, when [*M*] becomes small and gets close to the *K_r_* value (∼ 0.01 *nM* at the trough), the Michaelian term can be reduced to $\frac{V_{r}[M]}{2K_{r}}\;\sim \;\frac{\left[M\right]}{2}$ and the effective kinetics can be written as $\left(ks+\frac{1}{2})[M\right]$, which is equivalent to changing the *k_s_* value from 2 *h* ^−*1*^ to 2.5 *h* ^−*1*^ in the original model. The impact of such a change on the period length will certainly depend on the sensitivity of the model regarding the *k_s_* variation; our numerical results revealed that the inclusion of the Michaelis-Menten term (*R*-dependent mechanism), with those specific *K_r_* and *V_r_* values, only perturbs slightly the dynamics of the system, generating a first order effective PER translation kinetics very similar to that of the original model. This explains why for *nH* = 4 neither the period nor the amplitude changed drastically in the model considered in Fig. 2 *A*, as compared with the original [9]. For *nH* = 1, (Fig. 2 *B* and S2 Fig. *B* in S1 File), the [*M*] value (∼ 0.8 *nM* at the peak and ∼ 0.5 *nM* at the trough) is, at least, fifty times bigger than the *K_r_* value. In this case we could also consider that [*M* ] >> *K_r_* and infer that the effective translational kinetics is ultimately described by a first order law, very similar to the one presented in the original model. Therefore we would not expect any significant changes in the dynamical properties of the model considered in Fig. 2 *B* as compared with the original model. Although this seems to be true for the period length, our numerical results revealed that the oscillation amplitude changed significantly (∼ 17—19%) in our model as compared with the original one. This observation highlights, on one hand, that it is not always possible to intuitively predict the dynamical behavior of a system like the one presented here. On the other hand, this observation reveals that the changes in amplitude produced by the contribution of the *R*-dependent translation were indeed dependent on the Hill coefficient, with the differences being more evident for *nH* = 1 than for *nH* = 4. It is noteworthy that the Michaelis-Menten term, under the conditions of Fig. 2 *B*, impacts more on the peak-to-trough amplitude than on the period length.

To further study the changes in period and amplitude when the *R*-dependent mechanism has a bigger contribution to the overall translational process, in the next section we show a detailed numerical characterization of the dynamics of our model for different combinations of *k_s_*, *V_r_* and *K_r_* parameter values.

### Numeric characterization of oscillatory behavior as a function of the parameters related with PER translation

**Figure 3:**
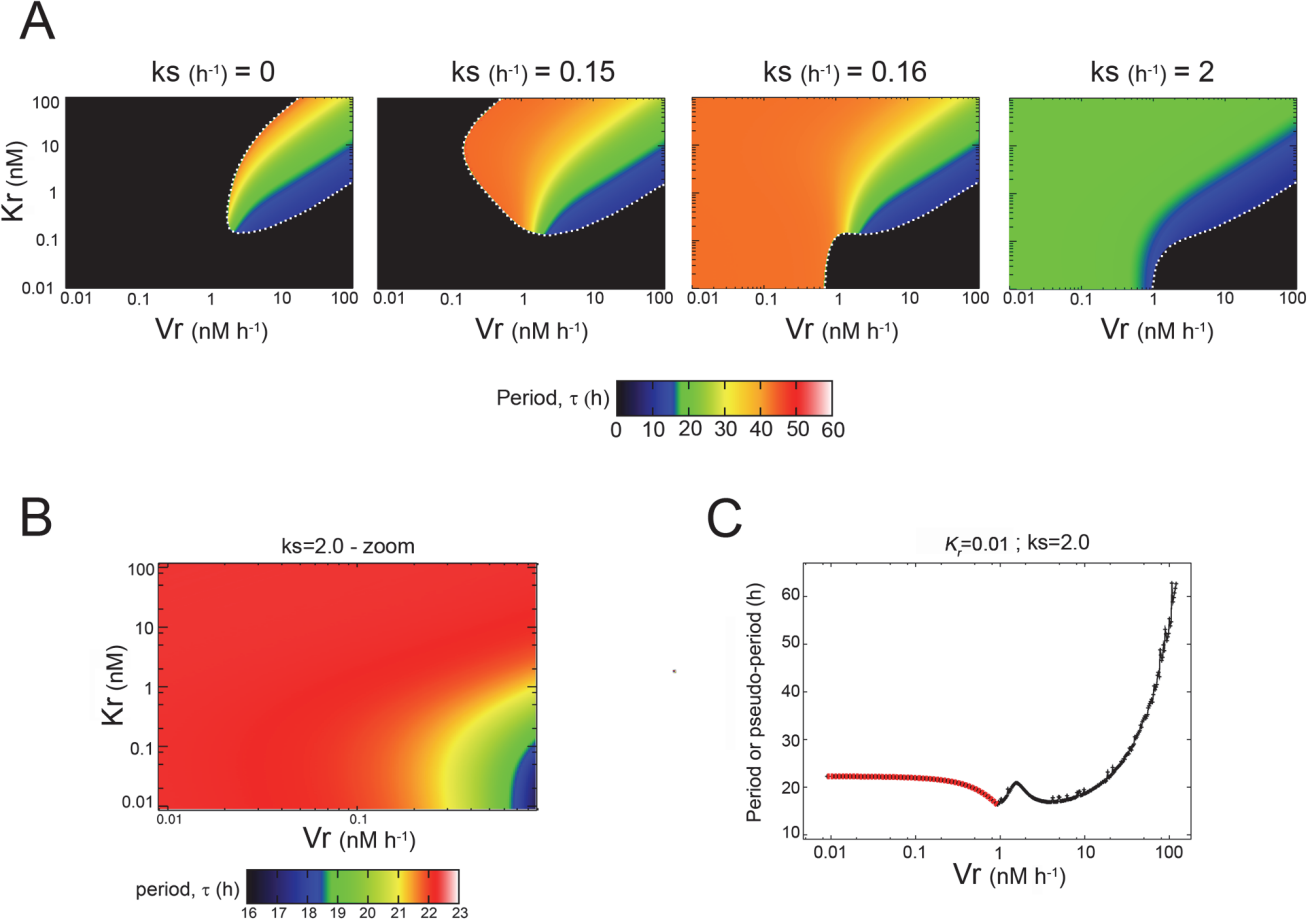
Dependence of the period (*τ*) with *k_s_*, *V_r_* and *K_r_*, for *nH* = 4. (*A*) The *τ*values are shown in pseudocolor within the *K_r_*-*V_r_*-parameter space for different values of the *k_s_* parameter from 0 (*left*) to 2 *h* ^−*1*^ (*right*). Black areas indicate zones in which there are damped oscillations. A white dotted line was added in order to emphasize the transition between the two dynamical behaviors observed in our model. Note the changes in the oscillatory domain and the *τ* length with *k_s_* (the color key for *τ* is at the bottom of the plots). (*B*) An inset of the *K_r_*-*V_r_*-space shown in panel *A* for *k_s_* = 2 *h* ^−*1*^ (*right*) shows the subtle changes in *τ*. Note that the key color was rescaled in order to display the differences in more detail. (*C*) Dependence of *τ* (*red line*) with *V_r_* for a fixed value of *K_r_* = 0.01 *nM*). Black line shows the pseudo-period value for damped oscillations. See the text for further details.

**Figure 4:**
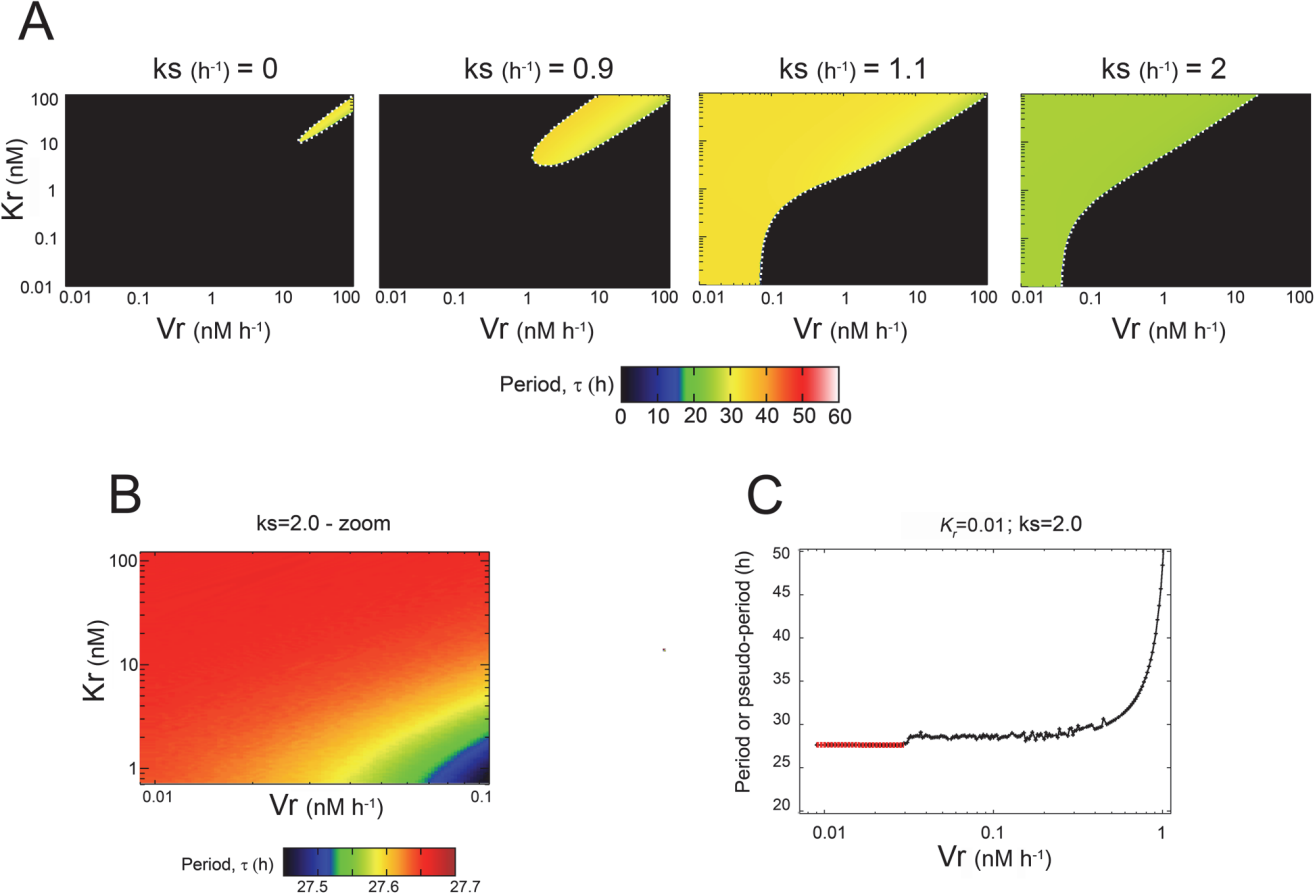
Dependence of the period (*τ*) with *k_s_*, *K_r_* and *V_r_* for *nH* = 1. (*A*) The *τ* values are shown in pseudocolor within the *K_r_*-*V_r_*-subspace for different values of the *k_s_* parameter, from *0* (*left*) to 2 *h* ^−*1*^ (*right*). Infradian (*τ* ≥ 28 *h*) are predominant for *k_s_* values < 2 *h* ^−*1*^. For each *k_s_* value, the range of *τ* values is narrower than those observed for *nH* = 4 (the color key for *τ* is at the bottom of the plot). Black areas indicate zones in which there are damped oscillations. A white dotted line was added in order to emphasize the transition between the two dynamical behaviors observed in our model. (*B*) An inset of the *K_r_*-*V_r_*-space shown in panel *A* for *k_s_* = 2 *h* ^−*1*^ (*right*) shows how *τ* changes within the circadian range; nevertheless the extent of *τ* change is smaller than that observed for *nH* = 4. Note that the key color was rescaled in order to display the differences in more detail (*C*) Dependence of *τ* (*red line*) with *V_r_* for a fixed value of *K_r_* = 0.01 *nM*. The black line shows the pseudo-period value for damped oscillations.

In order to study the dependence of the system dynamics on the translation-related parameters (*k_s_*, *V_r_* and *K_r_*), we numerically characterized the period (*τ*) and the amplitud (*a*) of the *per* mRNA (*M*) and total PER (*P*_t_) oscillations as functions of these parameters (Fig. 3, Fig. 4 and S5–S6 Fig. in S1 File).

For the cooperative model (*nH* = 4), Fig. 3 *A* shows the phase diagrams in the *K_r_*-*V_r_* subspace, in which the *τ* values are shown in pseudocolors and each diagram corresponds to a different *k_s_* value. The *k_s_* variations were introduced in order to analyze the relative importance of the *R*-independent translation versus the *R*-dependent mechanism. We have systematically varied the *k_s_* values ranging from 0 to 2 *h* ^−*1*^ and representative diagrams for specific *k_s_* values are presented in Fig. 3 *A*. For all the *k_s_* values considered, two major dynamic behaviors were observed depending on the *V_r_* and *K_r_* values: a region where the system oscillated in a sustained way (colored regions) and another region where the system decayed to a fixed point in an oscillatory manner (damped oscillations, black regions).

When the only mechanism involved in the PER translation was the *R*-dependent one (*k_s_* = 0 *h* ^−*1*^), we found a small domain within the *V_r_*-*K_r_* subspace in which the system oscillated self-sustainedly with different *τ*lengths, depending on the *V_r_*-*K_r_* parameter values. Circadian and close-to-circadian oscillations (green) emerged only in a region surrounded by the domains of ultradian (blue) and infradian (yellow to red) oscillations. In order to quantify the strength of saturation for the PER translation process taking place via the *R*-dependent mechanism, we numerically calculated the saturation index (*I*) values for each *K_r_*-*V_r_* combination within the oscillatory domain ([38], see details in the S1 File). S3 Fig. *A* in S1 File shows that a high proportion of the oscillatory domain presents small *I* values (between 0 and 0.5) and a small region exhibits large *I* values (between 0.5 and 0.8). However, the *I* values were never bigger than 0.8. We did not observe any correlation between the *I* values and the oscillation period. For *K_r_* >>> [*M*], the *I* values were small (i.e.: right-top corner of S3 Fig. *A* in S1 File) and the system with an *R*-dependent translation (*k_s_* = 0 *h* ^−*1*^) could trivially have the same behaviour than the original model introduced by Gonze ([9], *k_s_* = 2 *h* ^−*1*^; *V_r_* = 0 *nM h* ^−*1*^), as long as *V_r_* = *2K_r_* (green straight line plotted in S3 Fig. *A* in S1 File). Taken together, these results revealed that a PER translation process taking place exclusively via an *R*-dependent mechanism (Michaelis-Menten kinetics; *k_s_* = 0 *h* ^−*1*^) was sufficient to obtain self-sustained oscillations within a specific *V_r_*-*K_r_* domain; within that domain it was possible to obtain different degrees of saturation by tuning the *K_r_* and *V_r_* parameter values.

When *k_s_* > 0 *h* ^−*1*^, self-sustained oscillations emerged in *V_r_*-*K_r_* domains that increased with *k_s_* and spread out from the infradian oscillation region. Between *k_s_* = 0.15 *h* ^−*1*^ and 0.16 *h* ^−*1*^ the system exhibited a transition from a regime dominated by damped behavior to a regime dominated by self-sustained oscillations. In the latter regime, the shape of the oscillatory domain remained practically unchanged as *k_s_* increased, with only slight variations in the periodicity. We observed that as *k_s_* increased, the domains of infradian oscillations decreased in size. In particular for *k_s_* = 2 *h* ^−*1*^ the infradian region disappeared and the subspace was dominated by circadian and close-to-circadian behavior. Fig. 3 *B* is an inset of the diagram shown in Fig. 3 *A* for *k_s_* = 2 *h* ^−*1*^, which does not maintain the original color ranges exhibited in the corresponding full diagram because we wanted to highlight the subtle *τ* differences in more detail. This figure reveals that, for a fixed value of *K_r_*, an increase in the *V_r_* value produced a shortening of the *τ* length. This dependence of *τ* on the *V_r_* value was observed regardless of the *k_s_* values examined, which was clearly represented by the pseudocolor variation in each map of Fig. 3 *A*, going from red (long *τ* to blue (short *τ*). We did not observe, for any of the specific *k_s_* and *K_r_* values considered, any alteration of the pseudocolor gradient as *V_r_* grew (that is, *τ* codified in red > *τ* codified in orange > *τ* codified in yellow > *τ* codified in green > *τ* codified in blue). This information can be also observed in Fig. 3 *C* (red line), in which we have plotted the *τ* values as a function of the *V_r_* for a fixed value *K_r_* = 0.01 *nM*. This figure also includes the pseudo-period values of the damped oscillations (black line) present in the black region of the diagram shown in Fig. 3 *A* for *k_s_* = 2 *h* ^−*1*^ (see below). S3 Figs. *B–D* in S1 File show the *I* values within the *K_r_*-*V_r_*-diagrams for the other *k_s_* values shown in Fig. 3 *A*. We observed that the proportion of the oscillatory domain presenting large *I* values (i.e.: *I* ∼ 1) increased as we increased the *k_s_* value from 0.15 *h* ^−*1*^ (S3 Fig. *B* in S1 File) to 0.16 *h* ^−*1*^ (S3 Fig. *C* in S1 File), which means that the *R*-dependent translation becomes highly saturated in that region. This is likely to be related to the transition observed in the *K_r_*-*V_r_* diagrams, which changed from a regime dominated by damped behavior to a regime dominated by self-sustained oscillations (including the region of small *K_r_* and *V_r_* values). Nevertheless, when we further increased the *k_s_* value from 0.16 *h* ^−*1*^ (S3 Fig. *C* in S1 File) to 2 *h* ^−*1*^ (S3 Fig. *D* in S1 File) we observed that the region with small *I* values (between 0 and 0.5) becomes increasingly bigger. This is consistent with the fact that an increase of *k_s_* leads to an increase of the effective kinetics of PER translation, which reduces the overall mRNA concentration available to be translated by the *R*-dependent mechanism.

Fig. 4 shows a similar analysis but for the non-cooperative model (*nH* = 1). In this case (Fig. 4 *A*), for all the *k_s_* values studied, the domains where self-sustained oscillations emerged were very small compared with those obtained with the cooperative model (Fig. 3 *A*). Furthermore, in these domains only long circadian or infradian periods were observed. For *k_s_* = 2 *h* ^−*1*^, the whole oscillatory domain presented circadian oscillations with *τ* values ≈ 27—28 *h*: there was a decrease in the period length as *V_r_* increased, but for an identical increase in the *V_r_* parameter, the *τ* length decreased less when *nH* = 1 than when *nH* = 4 (compare Fig. 3 *B* and Fig. 4 *B*). Indeed, no ultradian periods were observed. Fig. 4 *C* also shows the changes in *τ* as *V_r_* increases, for a fixed value of *K_r_* = 0.01 *nM* (red line). Although the period appeared to be constant, it actually decreased slowly. The pseudo-period values (black line) corresponding to the damped oscillations (black region of the diagram shown in Fig. 4 *A* for *k_s_* = 2 *h* ^−*1*^) have also been included. The analysis of the *I* values for *nH* = 1 (S3 Fig. *E*–*F* in S1 File) is similar to that for *nH* = 4. The only difference is that, for *k_s_* = 0 *h* ^−*1*^ and *k_s_* = 0.9 *h* ^−*1*^, the system was not saturated regardless of the *K_r_*-*V_r_* combination tested. This means that the Michaelis-Menten kinetics can be approximated by a first order kinetics, and the effective translational kinetics (sum of *R*-dependent and *R*-independent) could be written as $k_{s}^{\prime }[M]$, with $k_{s}^{\prime }=(k_{s}+V_{r}/K_{r})$, which would give trivially the same result than the original model with a change in *k_s_*.

The pseudo-period values shown in Fig. 3 *C* and Fig. 4 *C* were included in order to show that beyond the self-sustained oscillatory domain the system displays transient damped oscillations before reaching the steady state. Using linear stability analysis we have confirmed the existence of Hopf bifurcations along the transition lines between the non-oscillatory (dampened) and oscillatory domains (white dotted lines in Fig. 3 *A* and Fig. 4 *A*; see the Supporting Information and S4 Fig. in S1 File). From the damped oscillations it was possible to measure pseudo-period values by numerically computing the average duration (time) between two consecutive peaks of the damped oscillations observed in PER concentration [39]. Near to the Hopf bifurcation point, these damped oscillations can exhibit pseudo-period values within the same range than the *τ* values corresponding to self-sustained oscillations on the other side of the transition lines.

The peak-to-trough amplitude (*a*) values for *M* and *P_t_* were also calculated, codified in a pseudocolor key, and represented in phase diagrams in the *V_r_*-*K_r_* parameter subspace, both for cooperative and non-cooperative models (S5 Fig. and S6 Fig. in S1 File, respectively). In these figures we show the *a* diagrams for *M* and *P_t_* at two different *k_s_* values, *k_s_* = 0 *h* ^−*1*^ and *k_s_* = 2 *h* ^−*1*^. We observed that, regardless of the value of the Hill coefficient considered, the amplitude range of *M* was larger for *k_s_* = 0 *h* ^−*1*^ than for *k_s_* = 2 *h* ^−*1*^, even when the latter produced a wider oscillatory domain. On the other hand, the amplitude range of *P_t_* for *nH* = 4 remained the same for both values of *k_s_*, whereas for the non-cooperative model (*nH* = 1) the amplitude ranges of *P_t_* changed slightly between the two *k_s_* values, being greater for *k_s_* = 0 *h* ^−*1*^. In addition, we also observed that a cooperative repression of *per* transcription always enlarged the oscillation amplitude, regardless of the values of *k_s_* explored, which is in agreement with the results shown previously in Fig. 2. As a final remark it is important to note that the results and conclusions showed in this section hold as long as the values for the rest of the parameters of our model are the same as those listed in S1 Table in S1 File.

### Characterization of the time lag between the per mRNA and PER protein expression profiles

**Figure 5:**
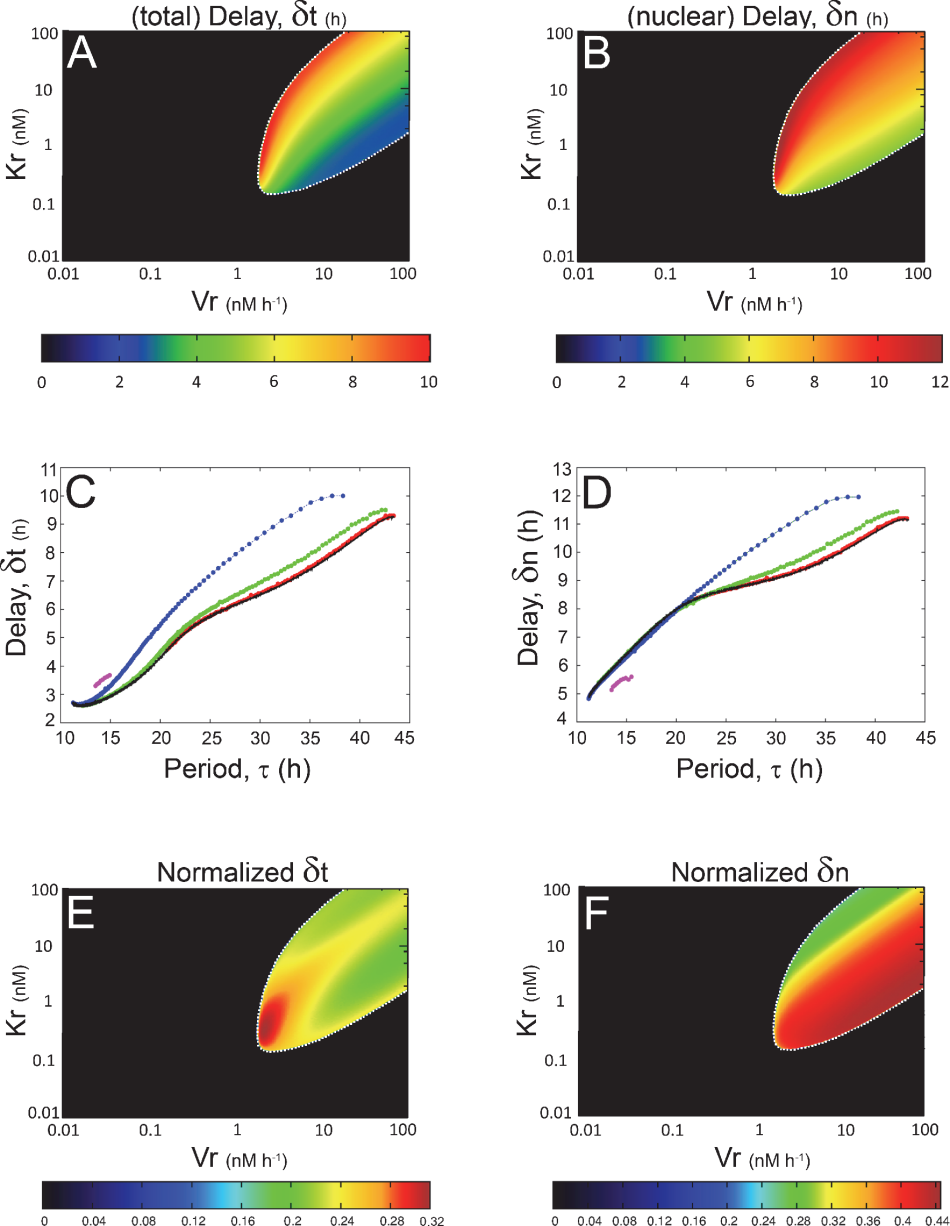
Analysis of the delays when PER translation is achieved by a single kinetic mechanism. Total delay (*A*) and nuclear delay (*B*) diagrams within the *V_r_*-*K_r_* parameter subspace, for a cooperative model in which *k_s_* = 0 *h* ^−*1*^. Total delay profile (*C*) and nuclear delay profile (*D*) diagrams as functions of the period *τ*. Each color line represents a fixed value of *K_r_*, as follows: red = 100 *nM*; green = 10 *nM*; blue = 1 *nM* and magenta = 0.14 *nM*. For a better comparison we have included the profile *δ_t_* versus *τ* for the model with a translational process achieved only by the *R*-independent mechanism (*V_r_* = *K_r_* = 0), which follows a first-order kinetics (black line). All these curves increase monotonically as *τ* increases. Figs. 5 *E* and *F* represent, respectively, the normalized total delay and nuclear delay diagrams within the *K_r_*-*V_r_*-subspace. The color key for each *K_r_*-*V_r_*-diagram is shown at the bottom of each plot. In those diagrams a white dotted line was added in order to emphasize the transition between the two dynamical behaviors observed in our model.

A still open question about the molecular circadian clock is how the delay (denoted by *δ*) between the expression of *per* mRNA and protein is achieved. Long time lags between the mRNA and protein expression profiles of PER have been observed experimentally: the phase of PER expression is delayed about 6–8 h relative to the mRNA [40–42]. This time lag was mainly associated with the stability of mRNA and proteins, post-translational modifications, dimerization processes, nuclear translocation, etc [42–45]. However, it was also suggested that the translational regulation may also be contributing to the observed delays [40, 46]. To test this hypothesis, we have computed the time delay (*δ*) between the maximum expressions of *per* mRNA and nuclear PER (denoted by *δ_n_*) and between the maximum expressions of *per* mRNA and total PER (denoted by *δ_t_*) obtained with our model, for different *k_s_*, *K_r_* and *V_r_* values. These *δ* values are represented in pseudocolor within the *K_r_*-*V_r_* subspace for the cooperative and non-cooperative cases (Fig. 5 and S8, S14 and S16 Figs. in S1 File).

We started by examining the delays obtained with the original model, which assumed a first-order kinetics for the translational process [9]. This condition was achieved in our model by setting *V_r_* = 0 *nM h* ^−*1*^, *k_s_* = 2 *h* ^−*1*^ and *nH* = 4. Under these conditions, the *δ_n_* value was about 8.39 *h* and the *δ_t_* value was about 5.11 *h* (S7 Fig. *A* in S1 File, arrows). S7 Fig. *A* in S1 File also shows a further characterization of the delays dependence on the *k_s_* parameter when the translational kinetics followed a first-order rate and *nH* = 4: a decrease of *k_s_* led to an increase of both *δ_n_* and *δ_t_* values. A similar profile was found for the variation of the oscillation period (*τ*) with *k_s_* (S7 Fig. *C* in S1 File). Furthermore, a monotonic relationship between delays and period was the prevailing trend observed for *nH* = 4 (S7 Fig. *E* in S1 File), in which the longer the period, the greater the delay. Minor exceptions to this behavior were found at each end of the *δ*_t_ profile (i.e.: for very short or very long periods), where the *δ_t_* values have changed little from one period to the other. We also observed a slowdown of the *δ_n_* profile for very long periods. For *nH* = 1, the period monotonically decreased with *k_s_*, just as for *nH* = 4 (S7 Fig. *D* in S1 File). Nevertheless, the relationship between delays and *k_s_* was not monotonic (S7 Fig. *B* in S1 File), which clearly impacted on the delays-versus-period profiles: delays passed through a maximum as a function of the period length (S7 Fig. *F* in S1 File).

**Figure 6:**
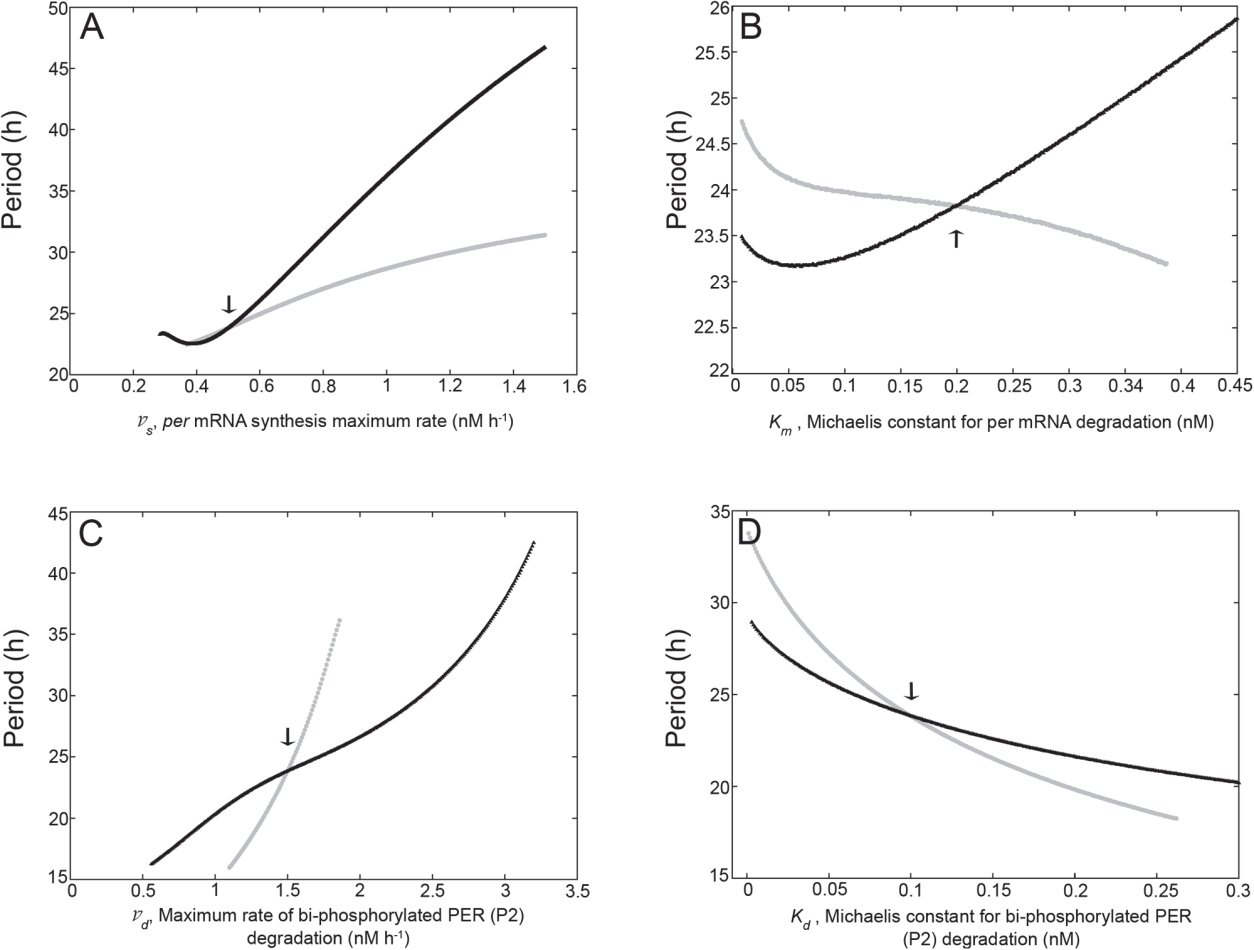
Profiles of the period (*τ*) as a function of several non-translation related parameters of the model. Dependence of the period (*τ*) on: the maximum rate of *per* mRNA synthesis, *v_s_* (*A*); the Michaelis constant for *per* mRNA degradation, *K_m_* (*B*); the maximum rate of PER degradation, *v_d_* (*C*) and the Michaelis constant for PER degradation, *K_d_* (*D*). We compared a model in which PER translation take place by an *R*-independent mechanism (first-order kinetics; *k_s_* = 1.48 *h* ^−*1*^; *K_r_* = 0 *nM* and *V_r_* = 0 *nM h* ^−*1*^; black curves) and a model in which PER translation take place by an *R*-dependent mechanism (Michaelis-Menten kinetics; *k_s_* = 0 *h* ^−*1*^; *K_r_* = 0.3 *nM* and *V_r_* = 2.09 *nM h* ^−*1*^; gray curves). Arrows indicate the values of the control parameters listed in S1 Table in S1 File.

When PER translation was achieved only through an *R*-dependent mechanism (i.e.: *k_s_* = 0 *h* ^−*1*^) and *nH* = 4, the delay values depended on the *V_r_* and *K_r_* values (Fig. 5 *A* and *B*). For a fixed value of *K_r_*, delays tended to decrease as *V_r_* grew (S9 Fig. *A* and *B* in S1 File, rhombs), which qualitatively resembled the dependence of delays with *k_s_* previously observed in Fig. 6 *A*. However, a change in the *V_r_* parameter produced smaller changes in the delays than those arising from similar changes in *k_s_* for the original model because the presence of the *K_r_* parameter in the Michaelis-Menten mechanism produced opposing effects on the delays, which tend to increase as *K_r_* grows (S9 Fig. *C* and *D* in S1 File, rhombs). Conversely, when *nH* = 1 the dependence of delays with *V_r_* or *K_r_* was not monotonic (S8 Fig. *A* and *B* in S1 File; see also S9 Fig. in S1 File, circles).

Figs. 5 *C* and *D* (see also S11 and S12 Figs., *B* and *C*, in S1 File) show the relationships between delays and period for *k_s_* = 0 *h* ^−*1*^ and *nH* = 4, which were qualitatively similar to those shown in S7 Fig. *E* in S1 File: the prevailing behavior observed was a monotonic relationship between delays and period, with slight deviations from this trend at each end of the *δ*_t_ profile and in the region corresponding to very long *τ* of the *δ*_n_ profile.

These results led us to ask how the delays arising from each translational mechanism considered here (i.e.: *R*-dependent vs *R*-independent) behave for a given (fixed) period of the molecular clock. In order to study this, we searched into the *K_r_*-*V_r_* parameter space shown in 5 Fig. (panels *A* and *B*) for a set producing oscillations with *τ* = 22.3 *h*, which is the period value reported for the original model [9]. For *nH* = 4 we found several combinations of the *V_r_* and *K_r_* parameter values that gave rise to oscillations with *τ* = 22.3 *h*, most of which yielded *δ_n_* and *δ_t_* values equal or longer than those obtained for the original model (S10 Fig. in S1 File). Indeed, for *δ_t_* this result was independent of the *τ* fixed for comparison (5 Fig. *C*; see also S11 Fig. *B* and *C* in S1 File). Notice that *δ_t_* becomes longer as the PER translation becomes more saturated (compare the *K_r_* isolines shown in 5 Fig. *C* with their *I* values shown in S3 Fig. *A* in S1 File).

Conversely, the *δ_n_* versus *τ* profile exhibited two different behaviors: for oscillations with periods longer than 20 *h* the *δ_n_* values obtained with an *R*-dependent translational mechanism were equal or longer than those obtained with an *R*-independent mechanism. On the other hand, for periods shorter than 20 *h* the *R*-dependent mechanism produced equal or shorter *δ*_n_’s than those obtained with an *R-*independent mechanism (5 Fig. *D*; S12 Fig. *B* and *C* in S1 File). In contrast, for *nH* = 1 the relationship between delays and period was not monotonic (S8 Fig. *C* and *D* in S1 File see also S13 Fig. *A* and *B* in S1 File) and delays obtained from a model with an *R*-independent mechanism of translation (i.e.: *V_r_* = 0 *nM h* ^−*1*^) were equal or longer than those obtained with an *R*-dependent mechanism (i.e.: *k_s_* = 0 *h* ^−*1*^), irrespectively of the *τ* fixed for comparison.

To further clarify the relationship between delays (*δ_t_* and *δ_n_*) and period (*τ*), we have normalized the delay values by the period length and characterized the normalized delay sensitivity regarding the *V_r_* and *K_r_* values (5 Fig. *E* and *F*). For *nH* = 4 this analysis revealed changes in the delays that were independent of the period length, or, in other words, exclusively dependent on the parameters *V_r_* and *K_r_*. This effect seemed to be enhanced by the cooperative repression of *per* transcription (i.e.: *nH* = 4), because for *nH* = 1 the normalized delay values were more homogeneous than for *nH* = 4 (compare 5 Fig. *E* and *F* with S8 Fig. *E* and *F* in S1 File).

From these observations we can conclude that a translational regulation process which generates a PER translation rate of Michaelis-Menten type (*R*-dependent) will contribute with equal or longer delays (*δ_t_* and *δ_n_*) to the molecular clock than a mechanism in which the PER synthesis rate grows linearly with the *per* mRNA concentration (*R*-independent), as long as *nH* = 4 and the *K_r_*-*V_r_* parameter combination generates oscillations with periods longer than 20 *h*. We also observed that a saturated kinetics of PER translation enlarges the *δ*_t_ values. These conclusions hold as long as the values for the rest of the parameters of our model are the same as those listed in S1 Table in S1 File.

Regarding the effect of the Hill coefficient (*nH*), we conclude that a non-cooperative transcriptional repression of *per* (*nH* = 1) tended to enlarge the delay values, irrespectively of the functional form of the translation kinetics considered here. For instance, for the original model (*K_r_* = 0 *nM*, *V_r_* = 0 *nM h* ^−*1*^, *k_s_* = 2 *h* ^−*1*^), a non-cooperative repression of *per* transcription resulted in longer delays (*δ_n_* = 8.77 *h* and *δ_t_* = 6.66 *h*) than those obtained with the cooperative model (*nH* = 4) (*δ_n_* = 8.39 *h* and *δ_t_* = 5.11 *h*) (S7 Fig. in S1 File, arrows) and the same qualitative result was obtained when the PER synthesis was achieved only by the *R*-dependent mechanism (S9 Fig. in S1 File). Furthermore, a non-cooperative transcriptional repression of *per* (*nH* = 1) tended to confine the diversity of delays observed within the *K_r_*-*V_r_* domain of self-sustained oscillations.

In addition, when we considered that PER translation was achieved by a combination of the two mechanisms proposed in our model (*R*-dependent plus *R*-independent), we observed that, by increasing *k_s_*, the delay distributions (both, the absolute and the normalized delays) became narrower than those observed for *k_s_* = 0 *h* ^−*1*^ (S14 and S15 Figs. in S1 File). This result was independent of the *nH* value considered here (S16 and S17 Figs. in S1 File). As a final remark we note that the observed nontrivial phase shifts (delays) require of the coupling of the various components of the circadian clock. If, for instance, a sinusoidal *per* mRNA were used as an input signal, the introduction of a Michaelian term would not *per se* lead to a change of the delays. This can be seen by considering the uncoupled Eq. (2) and noticing that the maxima and minima of *P_0_* coincide with the zeroes of the expression on the right-hand side, whose location is not changed by the presence of the Michaelian term.

Since assuming *nH* = 4 favored the periodic behavior of the system, facilitated the saturation of the R-dependent translational mechanism, increased the amplitude of mRNA oscillations and also increased the delays sensitivity to changes in the *V_r_* and *K_r_* values, we have continued our study considering that the repression of *per* transcription is a cooperative process.

### Influence of other parameters on the period of oscillations

Previous studies have reported that the phosphorylations, protein stability and nuclear translocations are major period determinants in the molecular circadian clock [42, 44]. For instance, Goldbeter [6] has shown that the maximum rate of PER degradation is a key control parameter of the model that he proposed. On the basis of these antecedents, we studied the sensitivity of the oscillatory behavior of our model to changes in their parameter values. In particular, we were interested in determining whether or not the kinetics of PER translation modifies the sensitivity of the oscillatory behavior to changes in parameter values. We compared, therefore, the influence of the parameter variation on *τ* when the PER translation is assumed to occur by a single kinetic pathway, either with Michaelis-Menten kinetics (*R*-dependent mechanism; *k_s_* = 0 *h* ^−*1*^) or with first-order kinetics (*R*-independent mechanism; *V_r_* = 0 *nM h* ^−*1*^ and *K_r_* = 0 *nM* ). In order to conduct the comparison between these two conditions we established two similar systems, that is, systems exhibiting sustained oscillations with the same *τ* value, whose only difference was the kinetics of PER translation. Starting from our ‘master’ model, we looked for these two similar systems by exploring and specifying either the *K_r_*-*V_r_* subspace in one case, or the *k_s_* subspace in the other, while the rest of the parameter values were maintained as presented in S1 Table in S1 File. In constant darkness, the *τ* observed in the *wild type* (WT) *Drosophila* is close to 24 *h* (i.e.: *τ* = 23.8 *h*, [25]). Consequently, we decided to set the *K_r_* and *V_r_* values in one case, and the *k_s_* in the other case, in such a way that the *τ* value, in each system, was 23.8 *h*.

Therefore, we compared a model in which PER translation takes place by an *R*-independent mechanism (first-order kinetics; *k_s_* = 1.48 *h* ^−*1*^; *K_r_* = 0 *nM* and *V_r_* = 0 *nM h* ^−*1*^); and a model in which PER translation takes place by an *R*-dependent mechanism (Michaelis-Menten kinetics; *k_s_* = 0 *h* ^−*1*^; *K_r_* = 0.3 *nM* and *V_r_* = 2.09 *nM h* ^−*1*^, white dot within the *K_r_*-*V_r_* diagram shown in S3 Fig. *A* in S1 File, *I* ∼ 0.72). After establishing either the *K_r_* and *V_r_* or the *k_s_* values, we maintained them fixed. Then, we proceeded to determine how *τ* is altered when each parameter (other than *k_s_*, *V_r_*, *K_r_* and *nH*) is varied (one at a time) by ± 100% of their original value (S1 Table in S1 File). Results are shown in Fig. 6 and S18 Fig. in S1 File.

The black curves in Fig. 6 and S18 Fig. in S1 File correspond to the model in which the PER translation occurred by an *R*-independent mechanism, while the gray curves correspond to the model in which the PER translation occurred by an *R*-dependent mechanism. Arrows in 6 Fig. indicate the initial values of the control parameters (S1 Table in S1 File). For all the cases shown in Fig. 6 we observed that the *τ* profiles changed significantly as a consequence of the functional form of the translation kinetics. On the other hand, the cases shown in S18 Fig. in S1 File did not exhibit significant changes between the two conditions tested here.

Fig. 6 *A* shows the dependence of *τ*on the Michaelis constant for *per* mRNA synthesis (*v*_s_). The model with an *R*-independent mechanism (black curve) exhibited a *τ* profile that initially decreased, *v*_s_ value increased. On the other hand, in the model with Michaelis-Menten kinetics (gray curve), the *τ* length increased monotonically as *v_s_* increased. Leloup and Goldbeter [38] defined two different types of sensitivity, one related to the extent of *τ* variation derived from changes in the control parameter (i.e.: influence of each parameter on the period), and the other related to the size of the oscillatory domain (i.e.: higher sensitivity when the window for oscillations is relatively narrow). For simplicity, here we named these two sensitivities as type I and type II, respectively. On the basis of this information we observed that an *R*-dependent translation decreased the type I sensitivity (i.e.: the slope of the black curve was steeper than the slope of the gray curve), but slightly increased the type II sensitivity (notice that the *v_s_* range in which oscillations emerged was quite narrow when the model assumed an *R*-dependent translation).

Fig. 6 *B* shows the dependence of *τ* on the Michaelis constant for *per* mRNA degradation (*K_m_*). When PER translation was achieved by an *R*-independent mechanism (black curve), the *τ* initially decreased, then passed through a minimum and finally increased as the *K_m_* value increased. When PER translation was achieved by an *R*-dependent mechanism (gray curve), the *τ* profile monotonically decreased as *K_m_* increased. In this case, an *R*-dependent mechanism decreased the type I sensitivity (the absolute value of the gray-curve slope is smaller than the black curve slope) and increased the type II sensitivity (notice that the black curve continues to grow for *K_m_* > 0.45 *nM*).

Thus, for the *v_s_* and *K_m_* parameters, which are important to establish the steady levels of the *per* mRNA, the two types of sensitivity changed similarly. Interestingly, when *v_s_* and *K_m_* are control parameters and the PER translation follows an *R*-dependent mechanism, the decrease of the type I sensitivity can be interpreted as if the synthesis and degradation of *per* mRNA would have partially lost power to influence the oscillation period.

Fig. 6 *C* shows the dependence of *τ* on the maximum rate of PER degradation (*v_d_*). The *τ* profile derived from a model with an *R*-independent mechanism of PER translation (first-order kinetics; black curve), monotonically increased as *v_d_* increased. This was in agreement with the profile reported by Goldbeter [6]. The *τ* values also rose as *v_d_* increased for a model with an *R*-dependent mechanism (gray curve), but the range of *v_d_* values for which sustained oscillations emerged was smaller and the slope of the curve was steeper than those observed with an *R*-independent translational mechanism. Therefore, a Michaelis-Menten based mechanism of PER translation (*R*-dependent) increased both type I and type II sensitivities.

Fig. 6*D* shows the dependence of *τ* on the Michaelis constant for PER degradation (*K*_d_). The shape of the *τ* profiles did not change significantly between the two cases compared here: both profiles showed that *τ* monotonically decreased as *K_d_* increased. Nevertheless, the *τ* profile derived from the model with an *R*-dependent translation (gray curve) exhibited a steeper slope than that obtained with the model where translation was achieved by an *R*-independent mechanism (black curve). In addition, the *K_d_* range for which sustained oscillations emerged was shorter in the model with an *R*-dependent translation than in the model with an *R*-independent translation. Thus, in this case, an *R*-dependent mechanism increased the two types of sensitivity considered here. The results of Fig. 6 *C* and *D* revealed that an *R*-dependent translation increased the sensitivity of the oscillation period to changes in the PER degradation process.

Overall, these results suggest that an *R*-dependent translation leads to a change in the global sensitivity of the system, in which the synthesis and degradation of *per* mRNA becomes less decisive, but the PER degradation becomes more significant on the determination of the oscillation period. It is important to notice that these conclusions hold as long as the rest of the parameters (that is, those parameters not considered as control parameters for each sensitivity analysis) maintain the values listed in S1 Table in S1 File.

### Testing our model with experimental results

After the exhaustive characterization of our model introduced in the previous sections, we proceeded to validate it by comparing its results with experimental data. In flies, Lim and coworkers [25] reported a *Drosophila* strain which has mutated a gene named *twenty four* (*tyf*). This gene codifies for a protein (TYF) involved in the regulation of *per* mRNA translation. It seems that TYF forms a complex with PABP (a RNA-binding protein which binds to the poly(A) tail of mRNAs) and ATAXIN-2 (ATX2), which ultimately interacts with the *per* transcript and activates PER translation in pacemaker neurons to sustain robust rhythms [25, 47, 48]. These results highlight the role of TYF-PABP-ATX2 complexes for the proper PER translation in the *Drosophila* neural clockwork. Importantly, *tyf* mutants expressed oscillating PER levels in lateral clock neurons but the peak-to-trough amplitude was lower than in WT flies. Furthermore, the period of behavioral rhythms in *tyf* mutants is longer than in WT flies. Since the TYF-PABP-ATX2 complex can associate with *per* mRNA and enhance the PER translation, we posit that TYF-PABP-ATX2 effects on PER translation kinetics could be well represented by a translational process occurring with a Michaelis-Menten kinetics. To test this hypothesis, we used a version of our model that considers that PER translation is only achieved by an *R*-dependent mechanism (Michaelis-Menten kinetics; *k_s_* = 0 *h* ^−*1*^). Here it is worth noting that, although in some conditions our model produces oscillations with *τ* = 22.3 *h* (Fig. 2 *A* and S1 *A* and S2 *A* Figs. in S1 File), this *τ* value does not correspond to the period observed in the WT *Drosophila* [25]. As shown in previous sections, we used this *τ* value only as a benchmark to conduct the characterization of our new version of the core clock model. Nevertheless, in order to see whether our simple model can account, at least qualitatively, for the experimental facts described above, it is important to show that it leads to circadian oscillations with *τ* = 23.8 *h*, which is the *τ* reported by Lim and coworkers for the (WT) *Drosophila* maintained in constant darkness [25].

**Figure 7:**
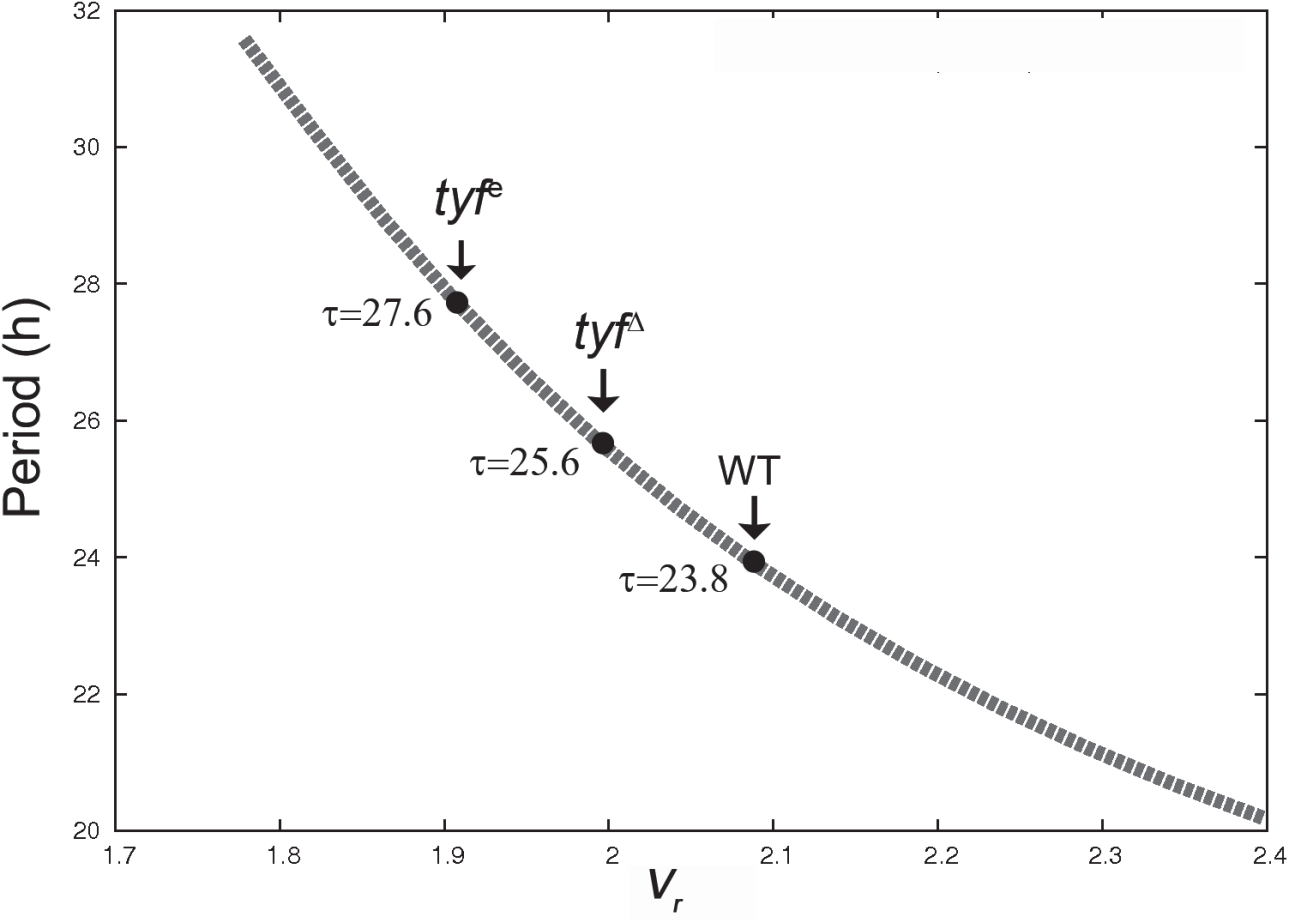
Comparison between theoretical and experimental results. The dashed gray line shows the dependence of *τ* with the *V_r_* parameter obtained with our model, for *k_s_* = 0 *h* ^−*1*^, *K_r_* = 0.3 *nM h* ^−*1*^ and *nH* = *4*. The periods of behavioral rhythms in wild type (WT) flies and *tyf* mutants (*tyf^e^* and *tyf* ^Δ^) are also indicated with black points and arrows, according to [25]. See the text for further details

**Figure 8:**
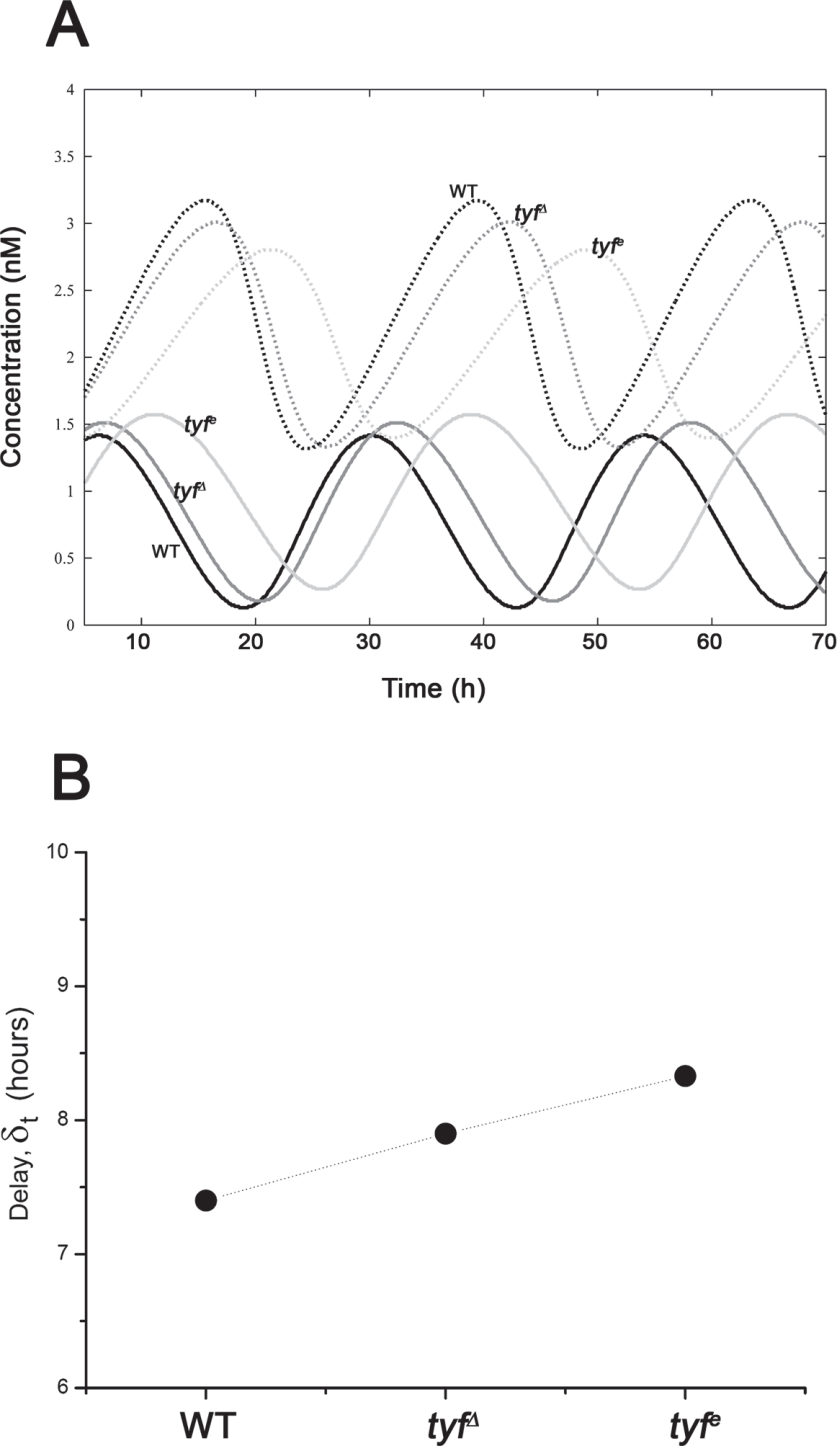
Circadian oscillations and delays in WT and *tyf* mutant flies. (A) Time evolution of the *per* transcript (*solid lines*) and total PER protein (*dashed lines*) in WT (*black*), *tyf* ^*Δ*^ (*dark gray*) and *tyf^e^* (*light gray*) flies. (B) Delay values (*δ_t_*) of each fly strain. Results obtained with our model for *k_s_* = 0 *h* ^−*1*^, *K_r_* = 0.3 *nM h* ^−*1*^ and *nH* = 4. Dotted line was plotted only for clarity purposes. See the text for further details.

Setting the translation-related parameters values at *k_s_* = 0 *h* ^−*1*^, *K_r_* = 0.3 *nM* and *V_r_* = 2.09 *nM h* ^−*1*^, (white dot within the *K_r_*-*V_r_* diagram shown in the S3 Fig. *A* in S1 File; *I* ∼ 0.72) and keeping the rest of the parameter values as listed in Table 1 in S1 File, our model exhibited sustained oscillations with *τ* = 23.8 *h* (Fig. 7 and Fig. 8 *A*). The *tyf* hypomorphic mutants can be modeled as a system with smaller values of the *V_r_* than WT flies because this parameter is related to the concentration of the TYF regulator (see discussion). Under these conditions, our model mimics the *τ* observed in the two *tyf* hypomorphic mutants reported by Lim and coworkers [25] (Fig. 7). Notice that *τ* became longer as the *V_r_* parameter decreased, which is qualitatively similar to the experimental results stated above. Another qualitative similarity between the theoretical and the experimental results was the decrease in the peak-to-trough amplitude of PER levels (Fig. 8 *A*) for the mutant flies. In addition, it is worth noting that our model predicted that the peak-to-trough amplitude *a* of the *per* mRNA for the mutants flies was lower than for WT flies, but the maximum levels of the *per* transcript were slightly higher (Fig. 8 *A*). By comparing the temporal evolution of the *per* mRNA and total PER obtained with our model for the parameter values used in Fig. 7, we could roughly predict the time delays *δ_t_* for WT and mutant flies (Fig. 8 *B*). We observed that the *δ_t_* (approximately 7.4 *h*) for the WT flies was shorter than those corresponding to the *tyf* ^*Δ*^ and the *tyf^e^* mutants (around 7.9 and 8.3 *h*, respectively, Fig. 8 *B*). These general and qualitative observations regarding the delay could be tested in future experiments.

## Discussion

In this work we studied the dynamical behavior of a minimal circadian clock model in which, depending on the translation-related parameter values, the PER translation can be achieved either by a single translation mechanism (either with a first-order or a Michaelis-Menten kinetics) or through a double-translational mechanism (the sum of the first-order and Michaelis-Menten kinetics terms). This ‘master’ model enabled us to study and compare the effects of different translational kinetics on the dynamics of the molecular clock. Changes in the kinetics of PER translation may be one important consequence derived from the PER translational regulation. Although recent experimental findings have highlighted the role of translational regulation in the circadian dynamics [25, 26], this level of regulation was not considered in previous theoretical studies of the circadian clock.

### PER translation by a single mechanism with Michaelis-Menten kinetics

Translational regulation is an important control point for shaping proteomes since it allows for more rapid changes in cellular protein concentrations than those achieved by transcriptional regulation [21, 49]. In mammalian cells, protein expression levels are primarily determined by regulation of translation and protein degradation [35]. In *Drosophila*, at least a 40% of the protein concentration is determined by these processes [50]. The kinetics of such biochemical processes are generally unknown and therefore, the choice of the kinetics laws and parameters to model these processes remains a challenge [44]. It is now recognized that the translation kinetics is a function of mRNA concentration as well as of other factors, such as the concentration of protein, tRNAs, ribosomes and translational regulators, among others. Similarly, protein degradation is also highly specific and tightly regulated. The involvement of multiple factors in the effective kinetics of both protein synthesis and degradation, suggests that these are nonlinear processes [34, 35]. In this regard, most of the previous models of the circadian clock assumed that the stationary concentration of total PER is mainly determined by its synthesis (considered as a first-order rate equation, dependent on the *per* mRNA levels) and degradation (modeled as a process that follows Michaelis-Menten kinetics) [6, 9, 29]. This description considers the nonlinearities related to the degradation process but not those related to translation. Although the assumption that the kinetics of PER synthesis follows a linear relationship with the amount of *per* mRNA has been satisfactory to model the molecular clock, we sought to determine how the clock dynamics is affected when nonlinearities in the translational process are included. Additionally, in recent years a growing number of reports have stressed the role of translational control mechanisms in the dynamics of circadian clocks [3, 20, 22, 23, 46]. In particular, several studies have pointed out that specific proteins act as enhancers of the *Drosophila* PER translation [25, 26, 47, 48]. According to these evidences, we considered that one simple way to take into account a nonlinear translational kinetics is by representing the PER translation as a process following a Michaelis-Menten kinetics. In addition to enhancing the global nonlinearity of the system without over-complicating the clock model, the saturable kinetics of Michaelis-Menten confers robustness to the biochemical oscillations by preventing the rates from increasing without limits [51]. Furthermore, a nonlinear saturable kinetics, such as the Michaelis-Menten, can roughly represent the kinetic consequence of a translational process regulated by one or multiple regulators acting either in a global and/or a specific fashion, which ultimately enhances the PER translation. Indeed, most models for the molecular clock have introduced nonlinearities (through a Michaelis-Menten kinetics) in other biochemical processes such as transcription, protein degradation and phosphorylation [43]. However the introduction of nonlinearities in the translational process has been rare. The only exception we know was the work of Kurosawa and Iwasa [52], which analytically proved (in circadian clock models with three or four variables) that the degree of saturation of enzyme kinetics affects the possibility of observing sustainable oscillations. They noticed that this effect varies systematically with the location of the reaction in the gene-protein network, and showed that large saturation indexes (*I*) for some reactions called *in-loop* (i.e.: PER translation, modification in the cytosol and transport to the nucleus) suppress the oscillation. Moreover, they also calculated the *I* values for each reaction step included in previous circadian clock models, and they showed that in those models the *I* values for in-loop reactions (like the PER translation) are small (between 0 and 0.5, see Fig. 5 within ref. [52]). We have numerically computed the saturation index for the PER translation reaction occurring through an *R*-dependent mechanism (see the Supporting Information and S3 Fig., *A* and *E*, in S1 File) and we also found that self-sustained oscillations emerged when the *I* values are small (between 0 and 0.5), which is consistent with the results of Kurosawa and Iwasa. Nevertheless, when *nH* = 4 our five-variable model showed that there is a small region within the *K_r_*-*V_r_* subspace in which self-sustained oscillations emerge even when the *I* values are larger than 0.5 (i.e.: *I* values between 0.5 and 0.8; S3 Fig. *A* in S1 File). This may suggest that in our five-variable model the saturation of PER translation does not necessarily imply an inhibition of the self-sustained oscillations.

Recently, the effect of Michaelis-Menten kinetics has been also discussed in the context of other cellular rhythms such as the activity of phosphofructokinase in glycolysis and cyclin-dependent kinases in the cell cycle [51], where it is shown and discussed how the Michaelis-Menten equation can be used to study the dynamical behavior of enzyme systems. Here we have shown that a PER translation process taking place exclusively via an *R*-dependent mechanism (Michaelis-Menten kinetics; *k_s_* = 0 *h* ^−*1*^) was sufficient to obtain self-sustained oscillations within a specific *V_r_*-*K_r_* domain (Fig. 3 *A*). Ultradian, circadian and infradian rhythms were observed, where the specific period, amplitude and delays depended on the specific values of the *V_r_* and *K_r_* parameters (Fig. 3 *A*, Fig. 6 and S4 Fig., *A* and *B*, in S1 File). These oscillatory properties also depended on the *nH* value (Fig. 4 *A* and S6, S8, S9 and S13 Figs. in S1 File) and other parameters of the model, therefore, the conclusions stated here hold as long as the values for the rest of the parameters of our model are the same as those listed in S1 Table in S1 File.

Extended clock models that incorporated the participation of *tim* and the formation of complexes between PER and TIM proteins revealed that the formation of the PER:TIM complexes favors sustained oscillations by introducing time delays in the negative feedback loop [7]. Qualitatively, this behavior is similar to that observed by a decrease of the Hill coefficient (*nH*) in core clock models, like the one presented here (S9 Fig. in S1 File). Recent work has highlighted the importance of studying how and to what extent the regulation of PER translation could contribute to those time delays observed experimentally [3]. Our study contributes to this point by showing that the specific kinetic function chosen to represent the translational kinetics would also affect significantly the time delays (Fig. 5 and S7–S17 Figs. in S1 File). Therefore, if the PER translational regulation involves a change in the translation kinetics, then it would also affect the time lags between the *per* transcript and protein expression profiles. Specifically, we showed that a PER translation taking place with a saturated Michaelis-Menten kinetics can contribute to the pace of the oscillator with longer delays than those produced by a first-order kinetics (Fig. 5), as long as *nH* = 4, the and the *K_r_*-*V_r_* parameter combination generates oscillations with periods longer than 20 *h*.

We remark that an increase in the delay follows a decrease in the Hill coefficient, independently of translation kinetics studied in this work (see S7 and S9 Figs. in S1 File). On the other hand, the presence of a Michaelian term in the translation could lead to an increase in the delays only if *nH* = 4, but not if *nH* = 1 (see Fig. 5 and S8 Fig. in S1 File). This suggest that the influence of the Hill coefficient on the delay goes beyond that of the translation kinetics. In addition, our results also indicate that the delay-shortening effect derived from an increase in the Hill coefficient can be compensated, at least in part, by changing the function describing the translation kinetics from first order to saturated Michaelis-Menten.

Previous studies showed that the dependence of *τ* on the PER degradation rate (*v_d_*) can adopt very different profiles depending on the kinetics of other processes, such as the *per* mRNA and protein degradation, PER phosphorylation-dephosphorylation and nuclear translocation [7, 53]. These results highlight the relevance of the nature of the kinetic functions describing the different biochemical processes on the clock properties. The results shown in Fig. 6 and S18 Fig. in S1 File revealed that the kinetic function describing the PER translational process also affected the dependence of *τ* on some of the non-translation related parameters of the model. Specifically, we observed that a saturable kinetics of PER translation changed the global sensitivity of the system, decreasing the sensitivity of the period to the synthesis and degradation of per mRNA, but increasing its sensitivity to PER degradation. Therefore, our results provide new information about how the kinetic laws of PER translation can modify the sensitivity of the oscillatory behavior of a core clock model to variations in their parameter values. These results have important implications if we assume that a regulatory process can act on the PER translation by changing its kinetic law. If that were the case, our results revealed that translational regulation may have strong implications for other biochemical process of the clock beyond translation itself. Another possibility would be to assume that the PER translational regulation proceeds without altering the kinetic laws but, instead, by changing the value of the translation-related parameters *V_r_* and *K_r_*. In this case, any alteration of the ‘normal’ regulation of PER translation must be reflected in such parameters. In this respect, the *Drosophila* TYF protein is a specific factor involved in the translational regulation of PER [25]. Hypomorphic mutations on the *tyf* gene result in a decreased expression of TYF, reducing the efficiency of PER translation. We have interpreted the effects of the *tyf* hypomorphic mutation as a decrease in the *V_r_* parameter because such parameter is related to (but not solely determined by) the concentration of specific translational regulators, like TYF. By making this qualitative interpretation of our model, we observed that the period of *tyf* hypomorphic mutants is shorter than the one observed for WT flies (Fig. 7), which qualitatively agrees with the experimental observations [25]. A similar interpretation of our model can be applied to other recent studies, which reported new proteins involved in the PER translational regulation, such as NAT1 and ATAXIN-2 [26, 48]. Moreover, by interpreting that the *tyf* hypomorphic mutations will result in a decrease of the TYF levels and, therefore, in a decrease of the *V_r_* parameter value of our model, we have made a qualitative prediction about the time delays expected for the flies carrying such mutations (Fig. 8).

### PER translation by two independent mechanisms

Two main mechanisms of translation initiation have been described in Eukaryotes: the canonical cap-dependent and the apparently less frequent cap-independent mechanisms [54]. Transcripts can hypothetically use both mechanisms, but it is unclear to what extent each of these pathways contributes to the total protein expression. Indeed, it is likely that the relative efficiency of one translational mechanism as compared to the other depends on each specific mRNA and the cellular conditions being studied, among other factors [54, 55]. Additionally, recent experimental studies have suggested that PER in *Drosophila* and PER1 in mammals are translated not only by a cap-dependent mechanism but also by a cap-independent pathway [26, 36, 56]. Because some of the regulators involved in these two translation initiation mechanisms may be different, it is likely that they present different kinetic laws and, therefore, can be modeled as two independent pathways. In this work we studied whether a circadian clock model including a PER translation process consisting of two kinetically different pathways, led to self-sustained oscillations, and how the oscillatory properties of this system were modulated by the relative contribution of those mechanisms. It is worth to emphasize here that our work is the first to systematically study the dynamics of a circadian clock model with an overall translation process derived from two translational mechanisms with different kinetics. Our results showed that it is possible to obtain self-sustained oscillations, with a plethora of periodicities (including the circadian one) and amplitudes that depend on the specific values of the *K_r_*, *V_r_* and *k_s_* parameters. For *nH* = 4, the *V_r_*-*K_r_* domains, in which self-sustained oscillations exist, became larger as *k_s_* increased, which reflected an increase of the robustness of the system when the *R*-independent mechanism became more important. A similar behavior was observed when *nH* = 1, except for *k_s_* = 2, which exhibited a slight decrease in the self-sustained oscillatory domain. Nevertheless, the diversity of observed periods and delays decreased as *k_s_* grew, for both *nH* = 4 and *nH* = 1. In addition, for a fixed set of *V_r_* and *K_r_* parameter values, an increase in *k_s_* produced a shortening of the oscillation period, which was revealed by the progressive disappearance of infradian periods in the *K_r_*-*V_r_* diagrams as *k_s_* increased (compare the *K_r_*-*V_r_* diagrams for *k_s_* = 0.16 *h* ^−*1*^ and *k_s_* = 2 *h* ^−*1*^ in Figs. 3 *A*).

It is well known that some drugs, like torin or rapamycin, cause inhibition of the canonical cap-dependent translation and shift translation toward a cap-independent activity [26, 28]. However, it is not clear yet whether and to what extent the cap-dependent and cap-independent initiation mechanisms of PER translation are relevant to the maintenance of the circadian period [26, 28]. In this context, mathematical models, like the one proposed here, may be useful to address these issues and to make reasonable predictions. For instance, it was shown that some translational regulators may be involved in the cap-independent translation of PER [28]. Therefore, it could be possible to draw a parallelism between the cap-independent/cap-dependent mechanisms and the *R*-dependent/*R*-independent mechanisms in our model, respectively.

This implies that the effect of torin or rapamycin on the cap-dependent translation may be represented in our model by abolishing or reducing the contribution of the *R*-independent mechanism to the overall translation, achieved by decreasing the value of the *k_s_* parameter. Under these conditions, at least one possible prediction emerging from our work is that treatment with those inhibitors will lengthen the period of the molecular clock (compare the *K_r_*-*V_r_* diagrams for *k_s_* = 0.16 *h* ^−*1*^ and *k_s_* = 2 *h* ^−*1*^ in Fig. 3 *A*) and it is expected to be experimentally tested in future works.

### Future directions and Concluding Remarks

More refined models of protein production could be also assumed in the future. For instance, an interesting approach could be to consider an oscillatory (circadian) regulation of PER translation, since recent studies in mammals revealed that translation is regulated by the circadian clock both globally (by translation initiation factors) and specifically (by RNA-Binding Proteins, micro RNAs and/or poly(A) tail length regulation) [20, 22, 27, 28].

It is worth noting that deterministic models of the circadian clock (as the one presented here) have been useful for the study of diverse aspects of circadian rhythmicity as long as the number of molecules involved in the oscillatory mechanism exceeds a few tens or hundreds of molecules. However, in some cellular conditions the number of molecules involved in the oscillatory mechanism may be small and deterministic models may reach their limits. In this situation the effect of the intrinsic molecular noise becomes important and it becomes necessary to resort to stochastic models. Previous stochastic models show that circadian oscillations are robust even at low numbers of molecules, although there is a threshold below which the noise obliterates the circadian rhythmicity [9, 57]. In addition, a variety of factors (such as the degree of cooperativity of repression, distance from the bifurcation point, the magnitude of the rate constants characterizing binding of the repressor to the gene, etc.) affects the robustness of the circadian oscillations in the presence of molecular noise. Thus, it would be interesting to explore the implications of assuming a nonlinear saturable kinetics for PER translation in stochastic models of the circadian clock, a problem that will be addressed in future works.

Our results have significant biological relevance because they characterized, for the first time, how different PER translational kinetics affects the generation and maintenance of circadian rhythms at molecular level. We showed that it is possible to modify significantly the period, amplitude, and delays of the self-sustained oscillations by changing the functional form of the PER translation kinetics, by changing the translation-related parameter values, or by considering the PER translation derived from two kinetically different pathways. We have interpreted the effective translational kinetics as the result of the regulation of PER synthesis, which might modify the translational functional term(s) and/or its kinetic parameters. Besides, our model provides results that are in qualitative agreement with experimental findings related to the PER translational regulation of the *Drosophila* molecular clock and supplies helpful information for interpreting and reassessing the significance of translational regulation in the molecular circadian clock.

## Supporting Information

S1 File (DOC)
