# Supplementary material for: Effects of Different PER Translational Kinetics on the Dynamics of a Core Circadian Clock Model
Source: PLoS One. 2015 Jan 21;10(1):e0115067. doi: 10.1371/journal.pone.0115067 (PMC4301915; doi:10.1371/journal.pone.0115067)
Supplement: S1 File — (DOC) [file pone.0115067.s001.doc]

**Supporting Information File S1**

**1.** **Supporting Table S1.**

Table S1: Parameters of the model schematized in Fig. 1. The results shown in our work (see article) have been obtained by numerical integration of Eqs. 1-5 for the following parameter values:

| Parameter | Definition | value* |
| --- | --- | --- |
| *v_s_* | *per* mRNA transcription rate | *0.5* |
| *K_i_* | Inhibition constant for repression of *per* mRNA transcription | *2.0* |
| *nH* | Hill coefficient | *1 or 4* |
| *K_m_* | Michaelis constant for *per* mRNA degradation | *0.2* |
| *v_m_* | Maximum rate of *per* mRNA decay | *0.3* |
| *k_s_* | rate constant for the R-independent translation | *0.0-2.0* |
| *V_r_* | Maximum rate of PER (P0) translation by the R-dependent mechanism | *0.01-100* |
| *K_r_* | Michaelis constant for PER (P0) translation by the R-dependent mechanism | *0.01-100* |
| *V_1_* | Maximum rate of unphosporylated PER (P0) phosphorylation | *6.0* |
| *K_1_* | Michaelis constant for unphosporylated PER (P0) phosphorylation | *1.5* |
| *V_2_* | Maximum rate of mono-phosphorylated PER (P1) dephosphorylation | *3.0* |
| *K_2_* | Michaelis constant for mono-phosphorylated PER (P1) dephosphorylation | *2.0* |
| *V_3_* | Maximum rate of mono-phosphorylated PER (P1) phosphorylation | *6.0* |
| *K_3_* | Michaelis constant for P1 phosphorylation | *1.5* |
| *V_4_* | Maximum rate of bi-phosphorylated PER (P2) dephosphorylation | *3.0* |
| *K_4_* | Michaelis constant for bi-phosphorylated PER (P2) dephosphorylation | *2.0* |
| *k_1_* | Rate constant for entry of bi-phosphorylated PER (P2) into the nucleus | *2.0* |
| *k_2_* | Rate constant for exit of bi-phosphorylated PER (P2) from the nucleus | *1.0* |
| *v_d_* | Maximum rate of bi-phosphorylated PER (P2) degradation | *1.5* |
| *K_d_* | Michaelis constant for bi-phosphorylated PER (P2) degradation | *0.1* |

***Units:** *K_i_*, *K_m_*, *K_1_*, *K_2_*, *K_3_*, *K_4_*, *K_d_* and *K_r_* are in nM; *v_s_*, *v_m_*, *V_1_*, *V_2_*, *V_3_*, *V_4_*, *v_d_* and *V_r_* are in nM h^-1^; *k_s_*, *k_1_* and *k_2_* are in h^-1^.

1. **Linear Stability Analysis**

To further characterize the transition between the two dynamical behaviors observed in our model, namely damped oscillations (fixed point dynamics) and self-sustained oscillations (limit cycle dynamics), we performed a linear stability analysis. Starting from the set of ordinary differential equations 1-5 that models the molecular circadian clock, we first obtained all the fixed points for a given set of parameter values. Next, we selected those with physical sense (real positive concentrations), evaluated the Jacobian matrix and calculated all its eigenvalues.

In Fig. S1 we plot the real part of the largest eigenvalue (denoted by λ) as a function of *V_r_*, for *nH*=4 and three different values of *K_r_*. Fig. S3 *A* shows the results obtained for *k_s_*= 0 h^-1^and Fig. S3 *B* for *k_s_*= 2 h^-1^. The regions with λ negative indicate a fixed point behavior. For *k_s_*= 0 h^-1^ and *K_r_*= 0.5 nM (Fig. S3 *A*, circles) we observed that transitions occur when the real part of a pair of complex-conjugates eigenvalues cross the imaginary axes twice, the first time from a negative to a positive value, indicating a Hopf bifurcation point at which the system undergoes a transition from damped oscillating dynamics to a self-sustained oscillatory regime. As *V_r_* increases, the system undergoes a reverse transition from the self-sustained to damped behavior. Similar results were observed for *K_r_*= 10 nM (Fig. S3 *A*, triangles), although we were unable to find the reserve transition, probably because we did not explore large enough values of *V_r_*. For small values of *K_r_*, (i.e., *K_r_*= 0.1 nM, Fig. S3 *A*, squares), the system always remains in the damped regimen, regardless of the value of *V_r_*. All the transition values obtained agree with those previously shown in Fig. 4 *A.*

For *k_s_*= 2.0 h^-1^ (Fig. S3 *B*), both the analysis and the interpretation are similar to those previously presented, with the observation that in all the cases analyzed there is only one transition from an oscillatory regime (small values of *V_r_*) to a damped one (large values of *V_r_*).

It should be underlined that a similar analysis for the non-cooperative case *nH*= 1 (data not shown), revealed very similar behavior, in agreement with the results shown in Fig. 5 *A*.

We have therefore corroborated that within the *V_r_-K_r_* subspace our equation system (Eq. 1-5) exhibited oscillations driven by stable limit cycles that emerge from a stable steady state by a Hopf bifurcation. The particular *V_r_-K_r_* subspace in which the limit cycle is observed depends on the particular values of *V_r_* and *K_r_* parameters and the *k_s_* and *nH* values considered.

1. **Saturation Index for the PER translation process.**

The saturation index (*I*) was introduced for the analysis of the clock models dynamics by Kurosawa and Iwasa  [52]. It quantifies the saturation strength of any reaction step ruled by a Michaelis-Menten kinetics and was defined as: *I* = *S*/(*K_m_*+*S*), where *K_m_* is the Michaelis constant (*K_r_* in our case) and *S* is the substrate concentration ([M] in our case) for the particular Michaelian process considered. This index ranges from 0 (null saturation) to 1 (full saturation). Based on the values of variables at equilibrium**,** Kurosawa and Iwasa examined the effect of saturation at each reaction step on the tendency of promoting/ suppressing  the self-sustained oscillations. They found that the saturation in any of the reactions included in the feedback loop (in-loop reactions: PER translation,  PER modification in the cytosol and transport to the nucleus) suppresses the system oscillation.

We used the *per* mRNA amplitude - i.e.: ([M] at the peak + [M] at the trough) /2] - to numerically compute the saturation indexes values for the R-dependent translation of PER within the oscillatory domains shown in Figures 3 and 4 A of our manuscript. Supplementary Figure S3 (Fig. S3) shows the dependence of the *I* values with the *K_r_*, *V_r_* and *k_s_* parameters, both for *nH* = 4 and *nH* = 1. Notice that when *k_s_* > 0 h^-1^, the *I* values reflected the saturation of the R-dependent PER translation, despite the fact that there is another PER translational mechanism contributing to the PER synthesis. In those cases, self-sustained oscillations emerged even when the R-dependent mechanism was strongly saturated (*I* = 1). These results were qualitatively similar when we used the *per* mRNA concentration at the peak or at the trough to compute numerically the I values within the oscillatory domains.

1. **Supporting figure caption**

**Figure S1**

System Dynamics. Repression of *per* transcription was assumed as a cooperative (***A*** and ***C***) or a non-cooperative (***B*** and ***D***) process. (***A*** and ***B***) Power spectrum analysis showed a marked periodicity for the system when a *R*-dependent mechanism is included with τ= 22.3 h and τ=27.7 h for the cooperative and the non-cooperative cases respectively. (***C*** and ***D***) Projection onto the (*M*, *P_t_*) plane showing the evolution towards a limit cycle trajectory starting from two different sets of initial conditions.

**Figure S2**

Comparison of the temporal evolution of the *per* mRNA (*M*) between the model including (*gray dotted line*) or not (*black solid line*) the *R*-dependent translation, for small values of *V_r_* and *K_r_* (*V_r_* = 0.01 nM h^-1^, *K_r_* = 0.01 nM, *k_s_* = 2 h^-1^). *(****A****)* Cooperative (*nH* = 4) or *(****B****)* Non-cooperative (*nH* = 1) repression of *per* transcription. For *nH* = 1, a small contribution of the *R*-dependent mechanism to the overall translation process produces a ~ 17-19 % of decrease in the peak-to-trough amplitude, while this remains unaffected for *nH* = 4 (note that the gray-dotted and black-solid lines are superimposed for *nH* = 4).

**Figure S3**

Dependence of the Saturation Index with *K_r_*, *V_r_* and *k_s_* values for both, *nH* = 4 (***A-D***) and *nH* = 1 (***E-H***). The *I* values (pseudocolor) range between 0 (null saturation) and 1 (full saturation). At the center of the figure is shown the color key for the *I* values exhibited in panels ***A, B, C, D, G*** and ***H***. Panels ***E*** and ***F*** include their own color keys. Panel ***E*** also includes an inset. The green straight line plotted in panel ***A*** indicates the isoline where *V_r_* = 2* *K_r_*, which corresponds to the cases in which our model is equivalent to the original model [9] when *K_r_* >> [M] (small *I* values) . The red arrow points towards a white dot within the oscillatory region shown in panel ***A***. The white dot indicates the *K_r_* - *V_r_* combination used in the simulations of Figures 6-8 (*k_s_* = 0 h−1 ; *K_r_* = 0.3 ; *V_r_* = 2.09 nM h−1 and ***I*** ~ 0.72). See the text for further details.

**Figure S4**

Real part of the largest eigenvalue (λ) obtained from the linear stability analysis of the equation system 1-5. The λ values are shown as function of the *V_r_* parameter. *(****A***) The λ obtained for different values of *V_r_* were plotted for three *K_r_* values (*squares* = *K_r_*= 0.1 nM; *triangles* = *K_r_* = 0.5 nM; *circles* = *K_r_* = 10 nM) and for *K_s_*= 0 h^-1^. (***B***). The λ obtained for same three *K_r_* values (*squares* = *K_r_*= 0.1 nM; *triangles* = *K_r_* = 0.5 nM; *circles* = *K_r_* =10 nM) , but for *k_s_* = 2 h^-1^. Lines connecting the points and the dotted line for λ= 0 were plotted only for clarity purposes.

**Figure S5**

Diagrams of the peak-to-trough amplitude a within the *V_r_* - *K_r_* parameter subspace, for *nH* = 4. (***A*** and ***C***) Amplitude values (a) for *per* mRNA (*M*) oscillations. (***B*** and ***D***) Amplitude values (*a*) for the total PER (*P_t_*) oscillations. Amplitude values are shown in pseudo-color for both *k_s_* = 0 h^-1^ (*top panels*) and *k_s_* = 2 h^-1^ (*bottom panels*), for the cooperative case (*nH* = 4). The color key for a is on the bottom of each plot. The white dotted line was added in order to emphasize the transition between the two dynamical behaviors observed in our model, namely damped oscillations (fixed point dynamics, black region) and self-sustained oscillations (limit cycle dynamics, colored regions).

**Figure S6**

Diagrams of the peak-to-trough amplitude (*a*) within the *V_r_-K_r_* parameter subspace, for *nH* = 1. (***A*** and ***C***) Amplitude values for  *per* mRNA (*M*) oscillations. (***B*** and ***D***) Amplitude values for the total PER (*P_t_*) oscillations. Amplitude values are shown in pseudo-color for both *k_s_* = 0 h^-1^ (*top panels*) and *k_s_* = 2 h^-1^ (*bottom panels*). The color key for *a* is on the bottom of each plot. The white dotted line was added in order to emphasize the transition between the damped (fixed point dynamics, black region) and self-sustained oscillations (limit cycle dynamics, colored regions).

**Figure S7**

Delay values (*δ*) between the *per* mRNA (*M*) and total PER protein expression profiles (*δt*) and between the *per* mRNA (*M*) and nuclear PER protein expression profiles (*δn*) when translation follows a first order kinetics (i.e.: R independent translational mechanism: *Vr* = 0 nM h^-1^, *Kr* = 0 nM). *δt* and *δn* values as function of the rate of PER synthesis (*ks*) for the R-independent mechanism for both, *nH* = 4 (***A***) and *nH* = 1 (***B***). The oscillation period (τ) monotonically decreases as *ks* increases for both *nH*=4 (***C***) and *nH* = 4 (***D***). Profiles of delays (*δ*) regarding the oscillation period (τ) reveals a monotonic or a non-monotonic dependence for *nH* = 4 (***E***) or for *nH* = 1 (***F***), respectively. Arrows indicates the *δ* and τ values for *ks* =2 h^-1^, which is the *ks* value reported in the original model [12].

**Figure S8**

Delay values (*δ*) between the *per* mRNA (*M*) and total PER protein expression profiles (*δt*) and between the *per* mRNA (*M*) and nuclear PER protein expression profiles (*δn*) when translation follows a Michaelis Menten kinetics (i.e.: R dependent translational mechanism = *ks* = 0 h^-1^) and *nH*=1. **(*A*** and ***B*)** show diagrams of *δt* and *δn* (respectively) within the *Vr-Kr* parameter subspace: *δt* ranges between 6.34 h and 7.7 h and *δn* ranges between 8.4 h and 9.8 h. (***C*** and ***D*)** show the profiles of *δt* and *δn* (respectively) regarding the oscillation period for five *Kr* conditions: cyan = 10.4 nM; magenta = 12.5 nM; blue = 25 nM; green = 50 nM; red = 100 nM. For comparison we have included the profiles shown in Figure S6, panel F, corresponding to the model with a first-order translational kinetics (black lines). (***E*** and ***F*)** show diagrams for the δt and δn values (respectively) normalized by the period length, within the *Vr-Kr* parameter subspace: normalized *δt* ranges between 0.22 and 0.25 and normalized *δn* ranges between 0.3 and 0.32 . The white dotted line was added in order to emphasize the transition between the damped (fixed point dynamics, black region) and self-sustained oscillations (limit cycle dynamics, colored regions).

**Figure S9**

Relationship between *δt* (***A*** and ***C***) and *δn* (***B*** and ***E***) regarding the *Vr (****A*** and***B****)* or the *Kr (****C***and***D****)* parameters when the PER translation is achieved only by a R-dependent mechanism (*ks* = 0 h^-1^). Delays values obtained when the transcriptional repression of *per* is a non-cooperative process (*nH* = 1, circles) are longer than those obtained with a cooperative model (*nH* = 4, rhombs). For panels ***A*** and ***B,*** each color represent a fixed value of *Kr*, as follows: blue = 16 nM; red = 20 nM; green = 25 nM; magenta = 32 nM. For panels ***C*** and ***D,*** each color represent a fixed value of *Vr*, as follows: cyan = 20 nM h^-1^ ; orange = 25 nM h^-1^; purple = 32 nM h^-1^; yellow = 40 nM h^-1^.

**Figure S10**

Relationship between *δt* (***A***) and *δn* (***B***) regarding the oscillation period (τ) when the PER translation is achieved only by a single mechanism, either a R-independent mechanism (*Vr* = 0 nM h^-1^, *Kr* = 0 nM, black squares) or a R-dependent mechanism (*ks* = 0 h^-1^, gray circles), for *nH*=4. Each circle corresponds to a single *Vr* and *Kr* combination and each square corresponds to a single value of *ks,* giving rise oscillations with τ around 22.3 h, which is the period obtained with the original model [12]. We observe that, within this period range, most of the delay values obtained by an R-dependent mechanism (gray circles) are equal or longer than those obtained for the original model [12] (arrow pointing the black square at *ks* = 2 h^-1^).

**Figure S11**

Profiles of *δt* versus τ for the four representative *ks* values shown in Figure 3***A*** (*nH* = 4). Panel ***A***,

***D***, ***G*** and ***J*** show the full range profile for *ks*= 0, 0.15, 0.16 or 2 h^-1^, respectively. Panels ***B***, ***E***, ***H*** and ***K*** corresponds to the inset 1 region shown within the full range plots. Panels ***C***, ***F***, ***I*** and ***L*** corresponds to the inset 2 region depicted within the full range plots, which include periods within

the circadian range. Panel ***A*** is the same plot shown in Figure 5***C*** (*ks*= 0 h^-1^), which was included in order to facilitate the comparison with the profiles for other *ks* values. Each color line represent a fixed value of *Kr*, as follows: red = 100 nM; green = 10 nM; blue = 1 nM; magenta = 0.14 nM and cyan = 0.1 nM . For a better comparison we have included the profile *δt* versus τ for the model with a translational process achieved only by the R-independent mechanism (*Vr* = 0 nM h^-1^, *Kr* = 0 nM), which follows a first-order kinetics (black line).

**Figure S12**

Profiles of *δn* versus τ for the four representative *ks* values shown in Figure 3***A*** (*nH* = 4). Panel ***A***,

***D***, ***G*** and ***J*** show the full range profile for *ks*= 0, 0.15, 0.16 or 2 h^-1^, respectively. Panels ***B***, ***E***, ***H*** and ***K*** corresponds to the inset 1 region shown in the full range plots. Panels ***C***, ***F***, ***I*** and ***L*** corresponds to the inset 2 region depicted in the full range plots, which include periods within the circadian range. Panel ***A*** is the same plot shown in Figure 5***D*** ( *ks* = 0 h^-1^), which was included in order to facilitate the comparison with the profiles for other *ks* values. Each color line represents a fixed value of *Kr*, as follows: red = 100 nM ; green = 10 nM; blue = 1 nM; magenta = 0.14 nM and cyan = 0.1 nM. For a better comparison we have included the profile *δt* versus tau for the model with a translational process achieved only by the R-independent mechanism (*Vr* = 0 nM h^-1^, *Kr* = 0 nM), which follows a first-order kinetics (black line).

**Figure S13**

Profiles of *δt* (***A***, ***C***, ***E*** and ***G***) or *δn* (***B***, ***D***, ***F***, ***H***) versus τ for the four representative *ks* values shown in Figure 4***A*** (*nH* = 1). Each color line represent a fixed value of *Kr*, as follows: red = 100 nM; green =50 nM; blue = 25 nM; magenta = 12.5 nM and cyan = 10.4 nM. For a better comparison we have included the profile of *δt* or *δn* versus tau for the model with a translational process achieved only by the Rindependent mechanism (*Vr* =0 nM h^-1^, *Kr* = 0 nM), which follows a first-order kinetics (black line).

**Figure S14**

Phase diagrams within the *Vr-Kr* parameter sub-space, showing the *δt* (***A-C***) or *δn* (***D-F***) values in pseudocolors, for *nH* = 4. For *ks*= 0.15 h^-1^, *δt* ranges from 2.6 h to 9.3 h (***A***) and *δn* ranges from 4.8 h to 11.3 h (***D***). For *ks*= 0.16 h^-1^, *δt* ranges from 2.6 h to 9.3 h (***B***) and *δn* ranges from 4.9 h to 11.3 h (***E***). For *ks*= 2 h^-1^, *δt* ranges from 2.9 h to 5.2 h (***C***) and *δn* ranges from 5 h to 8.4 h (***F***). The color key for each diagram is on the bottom of each plot. The white dotted line was added in order to emphasize the transition between the damped (fixed point dynamics, black region) and self-sustained oscillations (limit cycle dynamics, colored regions).

**Figure S15**

Phase diagrams within the *Vr-Kr* parameter sub-space showing the normalized *δt* (***A-C***) or the normalized *δn* (***D-F***) values in pseudocolors, for *nH* = 4. For *ks*= 0.15 h^-1^, the normalized *δt* ranges from 0.19 to 0.31 (***A***) and the normalized δn ranges from 0.23 to 0.44 (***D***). For *ks* = 0.16 h^-1^, the normalized *δt* ranges from 0.19 to 0.3 (***B***) and the normalized *δn* ranges from 0.25 to 0.44 (***E***). For *ks* = 2 h^-1^, the normalized *δt* ranges from 0.19 to 0.25 (***C***) and the normalized δn ranges from 0.35 to 0.44 (***F***). The color key for each diagram is on the bottom of each plot. The white dotted line was added in order to emphasize the transition between the damped (fixed point dynamics, black region)

and self-sustained oscillations (limit cycle dynamics, colored regions).

**Figure S16**

Phase diagrams within the *Vr-Kr* parameter sub-space showing the *δt* (***A-C***) or *δn* (***D-F***) values in pseudocolors, for *nH* = 1. For *ks* = 0.9 h^-1^, *δt* ranges from 6.4 h (***A***) to 7.7 h and *δn* ranges from 8.4 h to 9.8 h (***D***). For *ks* = 1.1 h^-1^, *δt* ranges from 6.4 h to 7.8 h (***B***) and *δn* ranges from 9.1 h to 9.8 h (***E***). For *ks* = 2 h^-1^, *δt* ranges from 6.3 h to 6.7 h (***C***) and *δn* ranges from 8.2 h to 8.8 h (***F***). The color key for each diagram is on the bottom of each plot. The white dotted line was added in order to emphasize the transition between the damped (fixed point dynamics, black region) and self-sustained oscillations (limit cycle dynamics, colored regions).

**Figure S17**

Phase diagrams within the *Vr-Kr* parameter sub-space showing the normalized *δt* (***A-C***) or the normalized *δn* (***D-F***) values in pseudo-colors, for *nH* = 1. For *ks* = 0.9 h^-1^, the normalized *δt* ranges from 0.22 to 0.25 (***A***) and the normalized δn ranges from 0.28 to 0.32 (***D***). For *ks* = 1.1 h^-1^, the normalized *δt* ranges from 0.22 to 0.25 (***B***) and the normalized δn ranges from 0.27 to 0.32 (***E***). For *ks* = 2 h^-1^, the normalized *δt* ranges from 0.23 to 0.24 (***C***) and the normalized *δn* ranges from 0.30 to 0.32 (***F***). The color key for each diagram is on the bottom of each plot. The white dotted line was added in order to emphasize the transition between the damped (fixed point dynamics, black region) and selfsustained oscillations (limit cycle dynamics, colored regions).

**Figure S18**

Profiles of the period (τ) as a function of several non-translation related parameters of the model.

Dependence of the period (τ ) on: Maximum rate of *Per* mRNA decay, *v_m_* , (***A***); Inhibition constant for repression of *Per* mRNA transcription, *K_i_*, (***B***); Maximum rate of unphosphorylated PER phosphorylation, *V_1_* , (***C***); Michaelis constant for unphosphorylated PER phosphorylation, *K_1_* , (***D***); Maximum rate of mono-phosphorylated PER desphosphorylation, *V_2_* , (***E***); Michaelis constant for mono-phosphorylated PER desphosphorylation, *K_2_* , (***F***); Maximum rate of mono- phosphorylated PER phosphorylation *V_3_* , (***G***); Michaelis constant for mono-phosphorylated PER phosphorylation, *K_3_* , (***H***); Maximum rate of bi-phosphorylated PER desphosphorylation, *V_4_* , (***I***); Michaelis constant for bi-phosphorylated PER desphosphorylation, *K_4_* , (***J***); Rate cosntant for entry of bi-phosphorylated PER into the nucleus, *k_1_*, (***K***); Rate cosntant for exit of bi-phosphorylated PER from the nucleus, *k2*, (***L***). We compared a model in which PER translation take place by an *R*-independent mechanism (first-order kinetics; *k_s_* = 1.48 h^−1^, *K_r_* = 0 nM and *V_r_* = 0 nM h^−1^; black curves) and a model in which PER translation take place by an *R*-dependent mechanism (Michaelis Menten kinetics; *k_s_* = 0 h−1, *K_r_* = 0.3 nM and *V_r_* = 2.09 nM h^−1^; gray curves). The τ – profiles did not exhibit significant changes between the two conditions tested here.

**5. Supporting figures**

**Figure S1.**

**
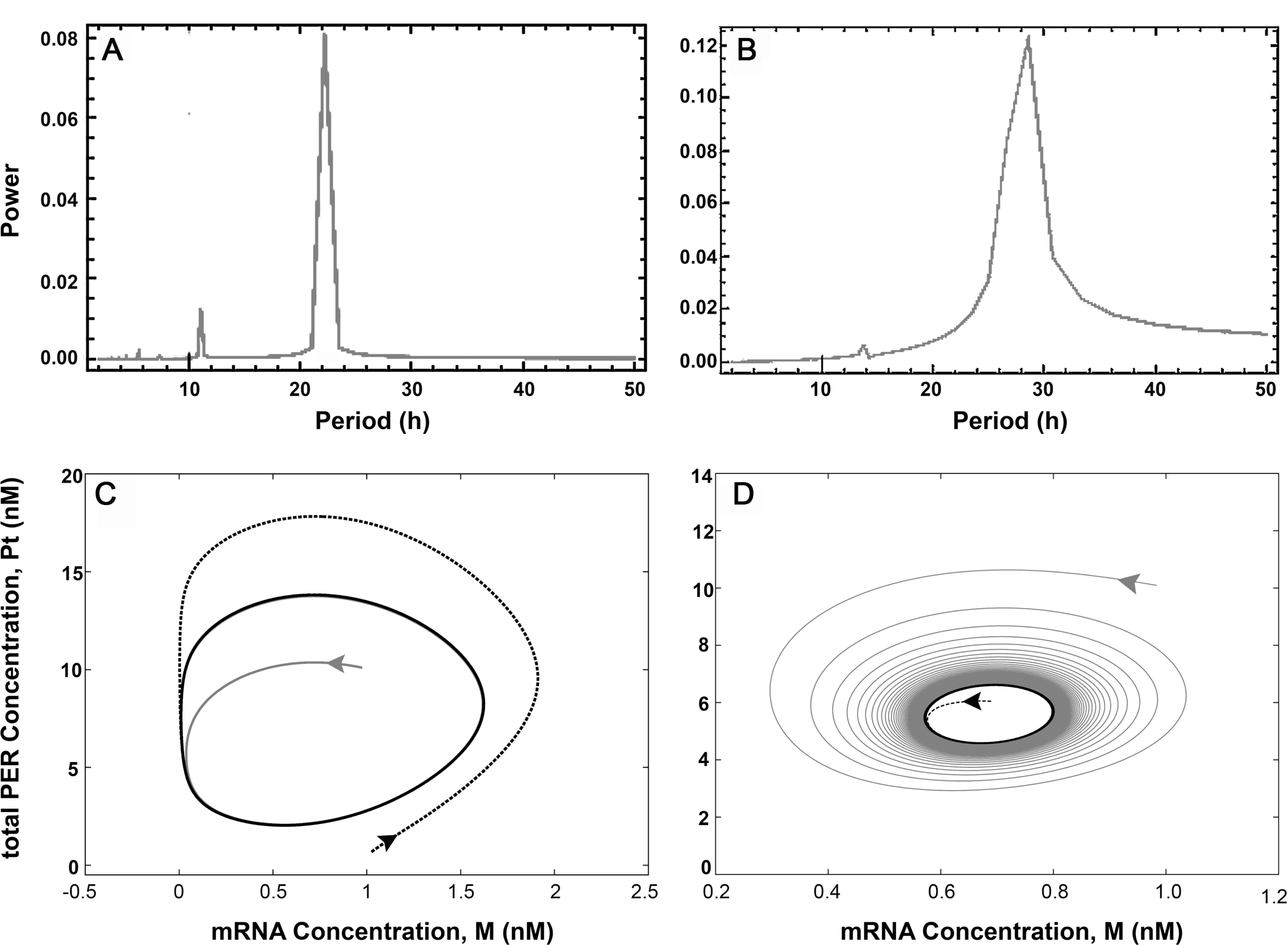
**

**Figure S2**

**
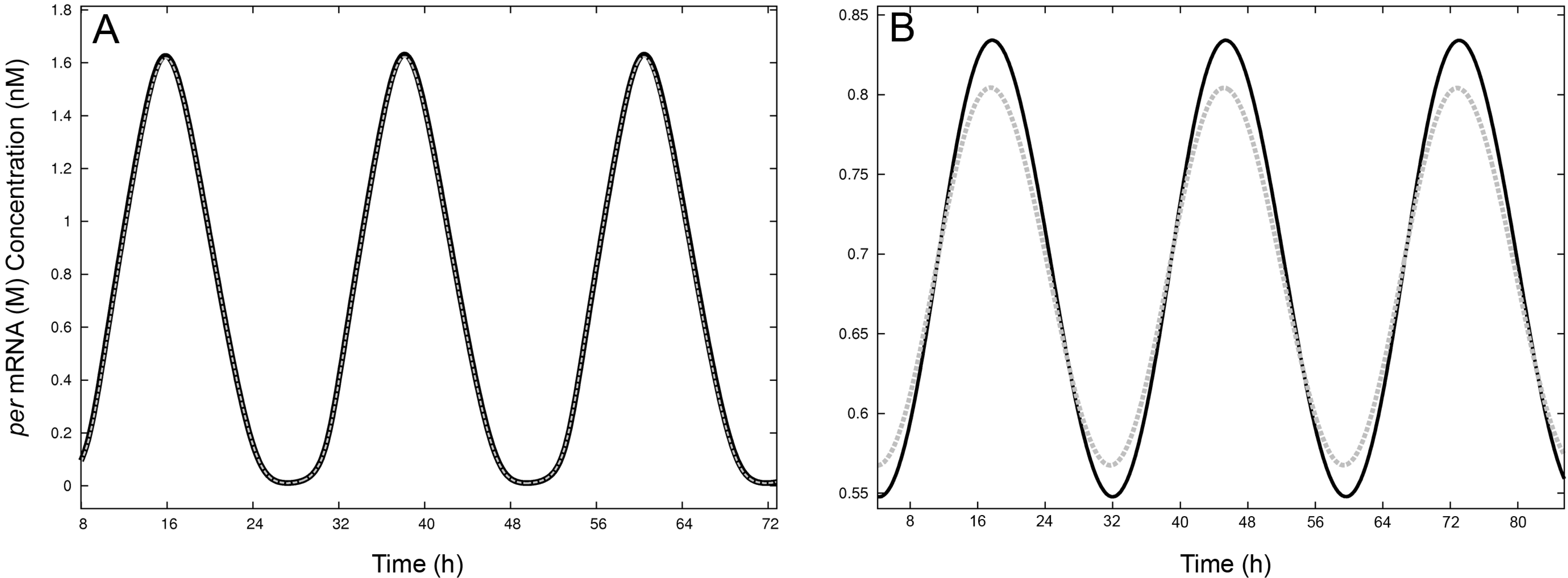
**

**Figure S3**

**
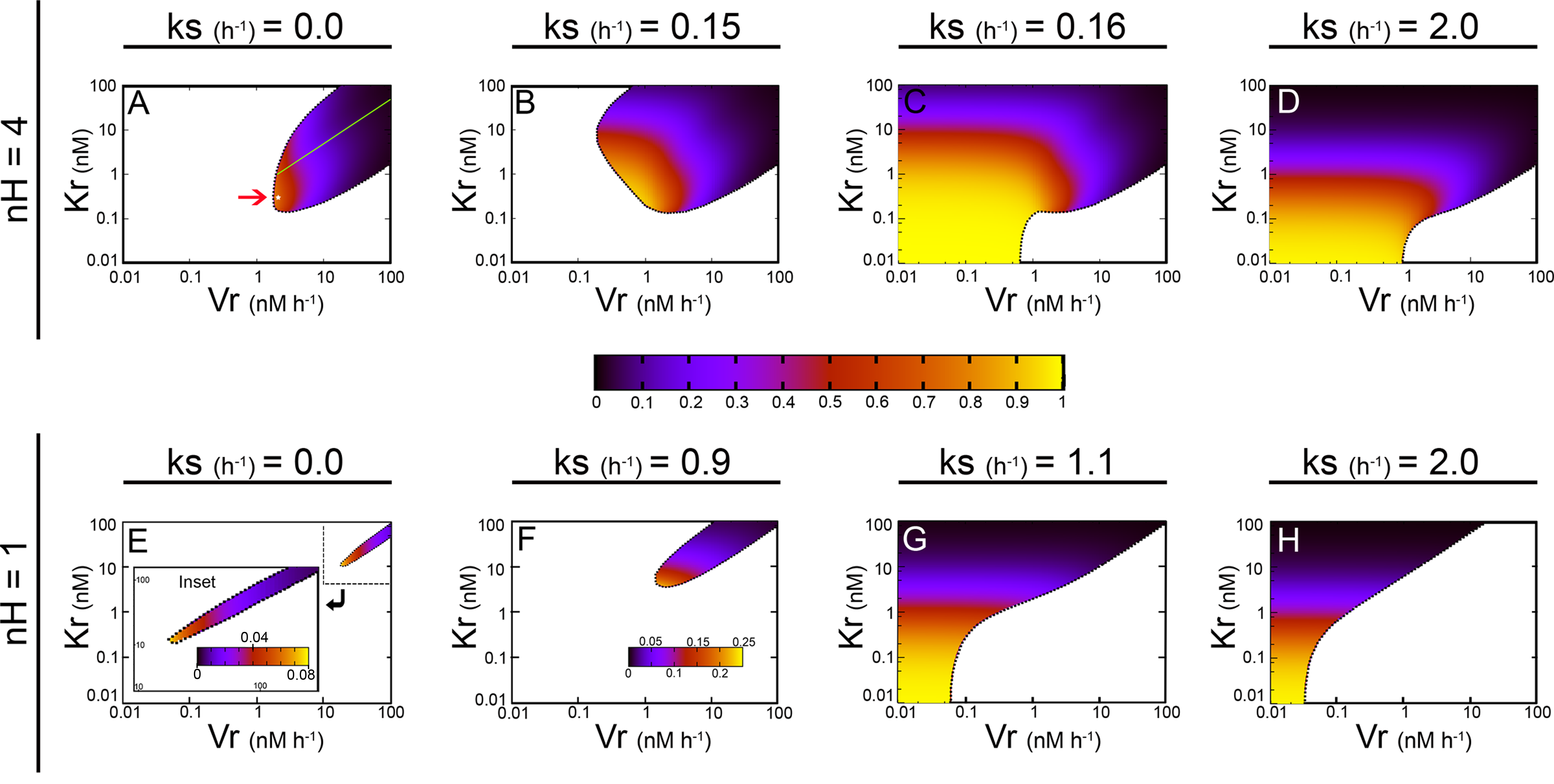
**

**Figure S4**

**
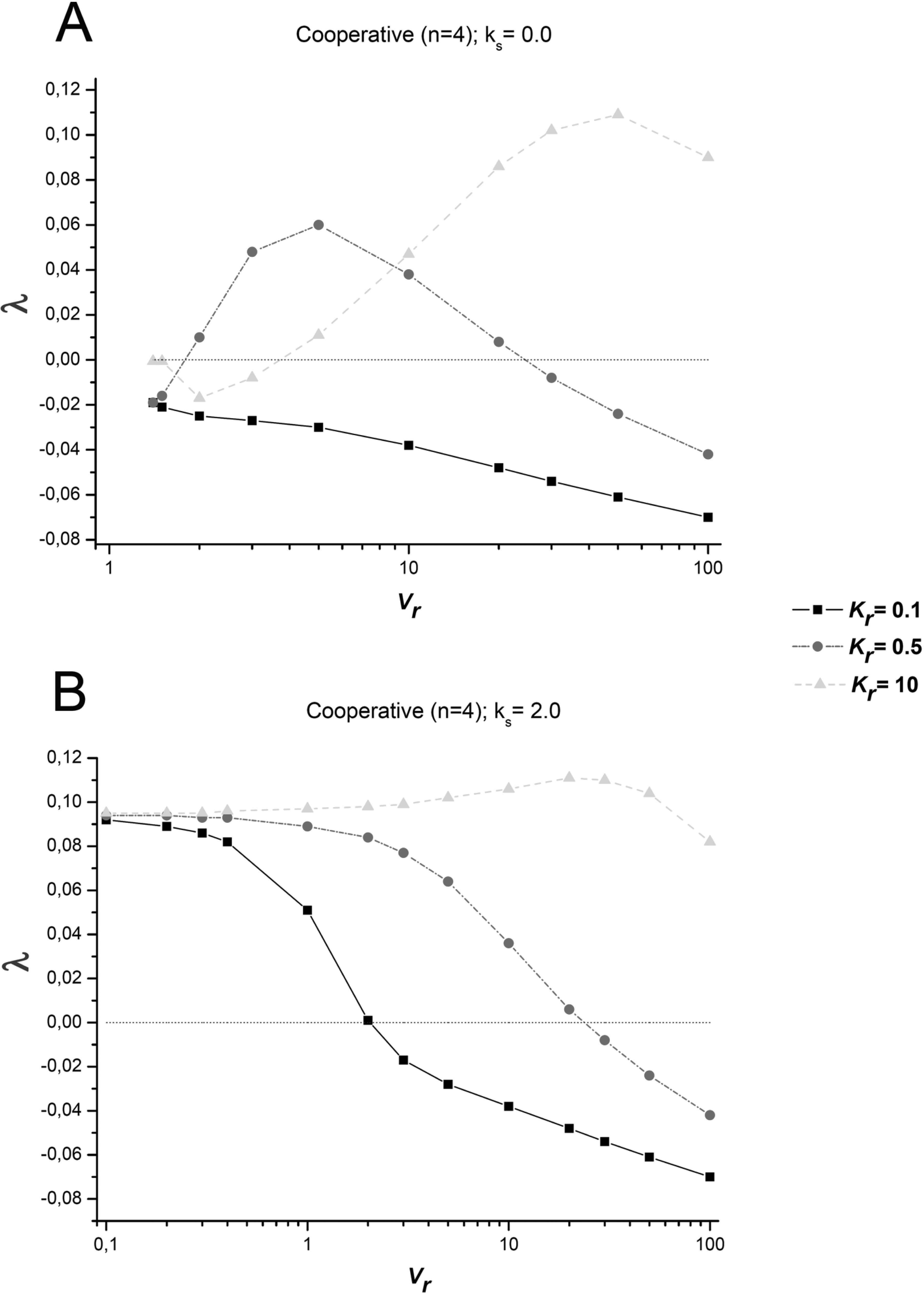
**

**Figure S5**

**
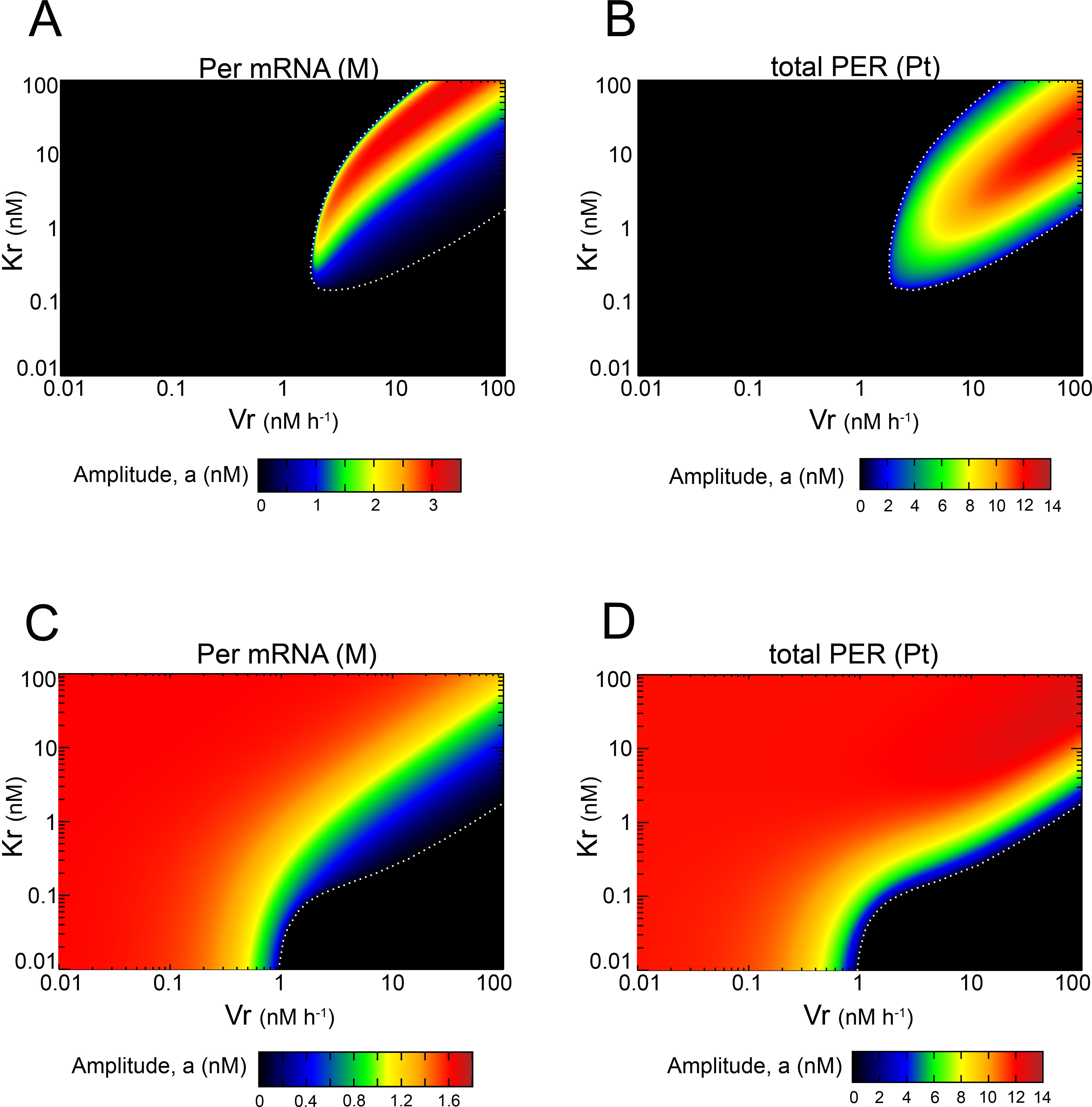
**

**Figure S6**

**
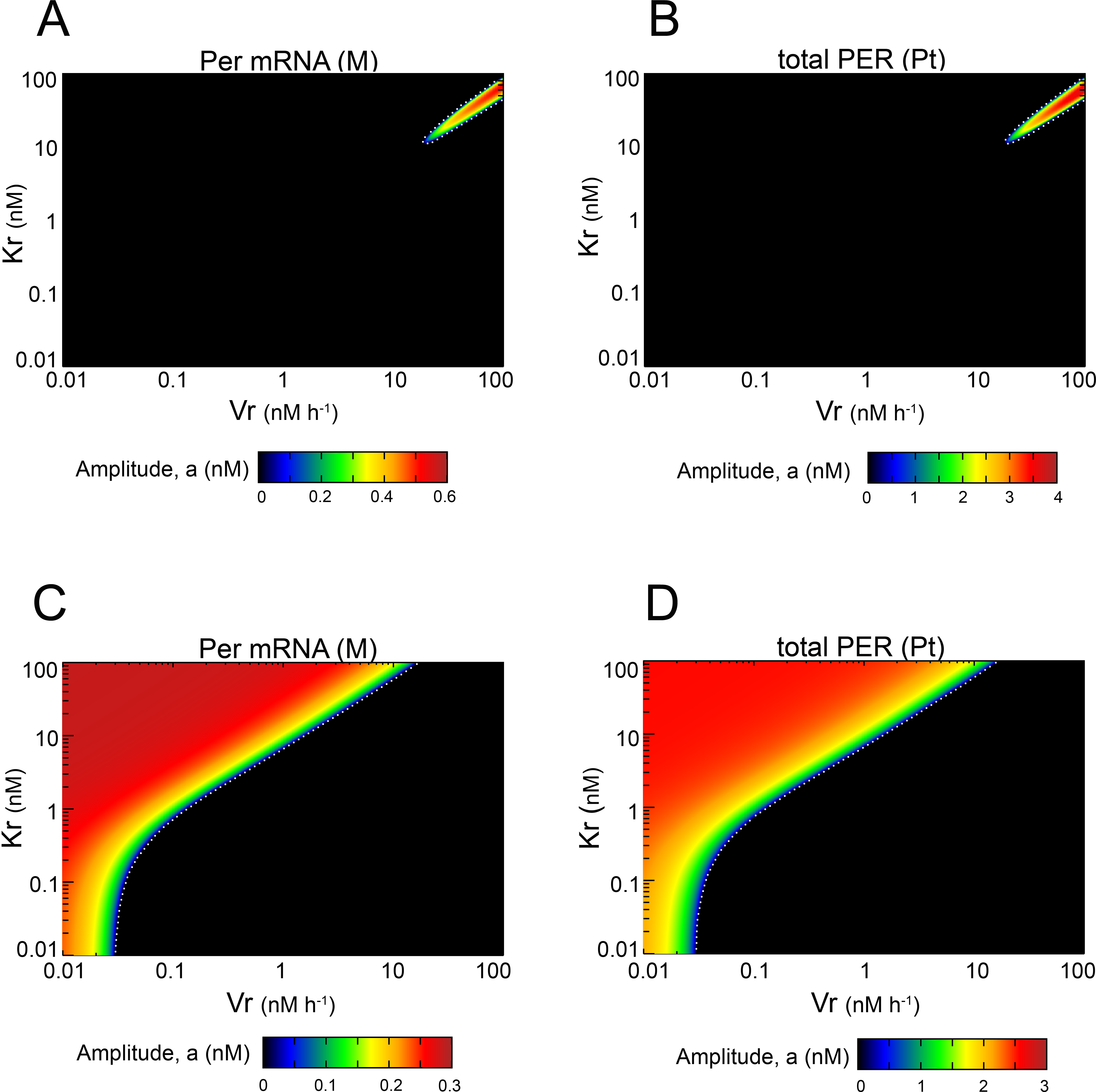
**

**Figure S7**

**
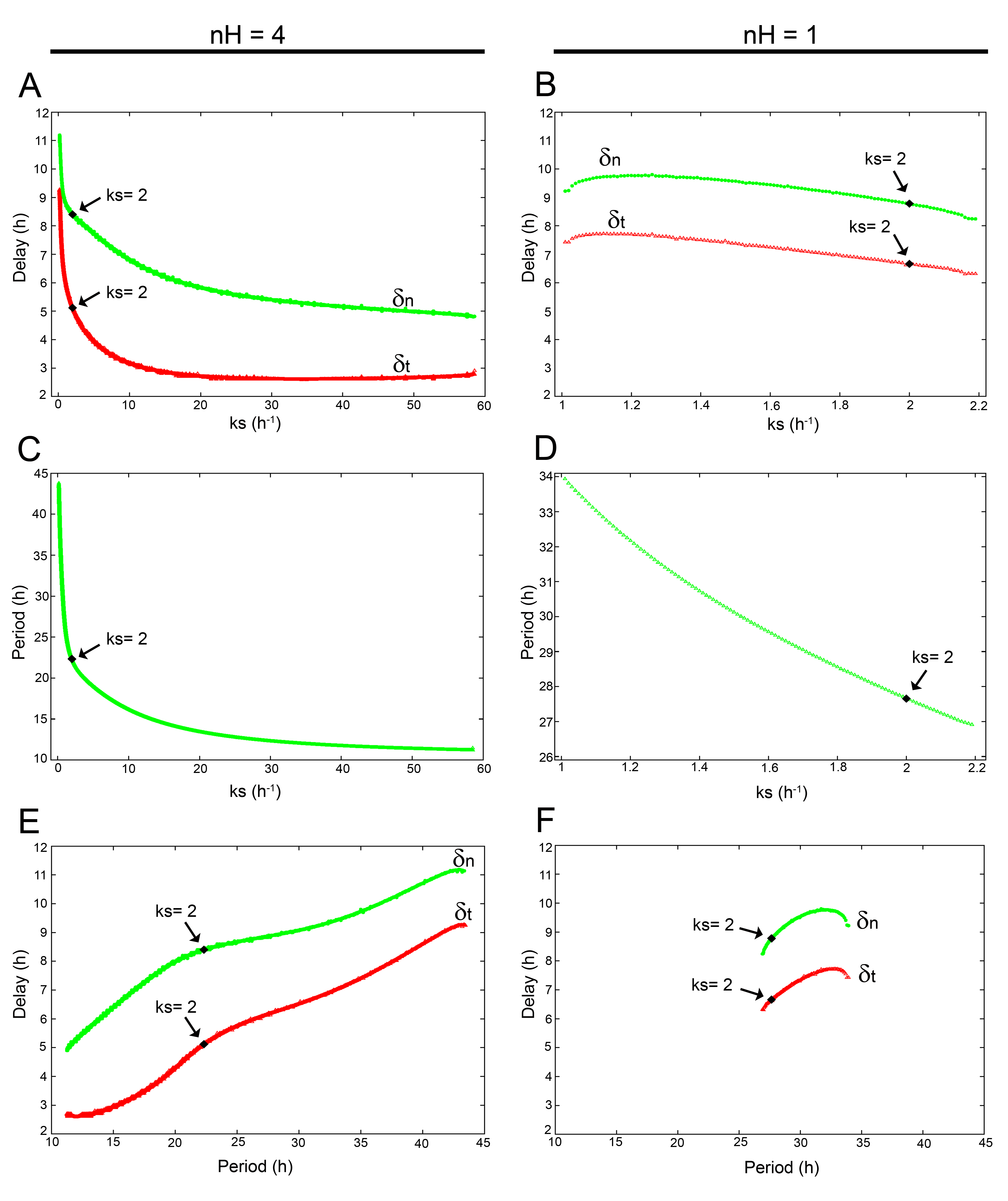
**

**Figure S8**

**
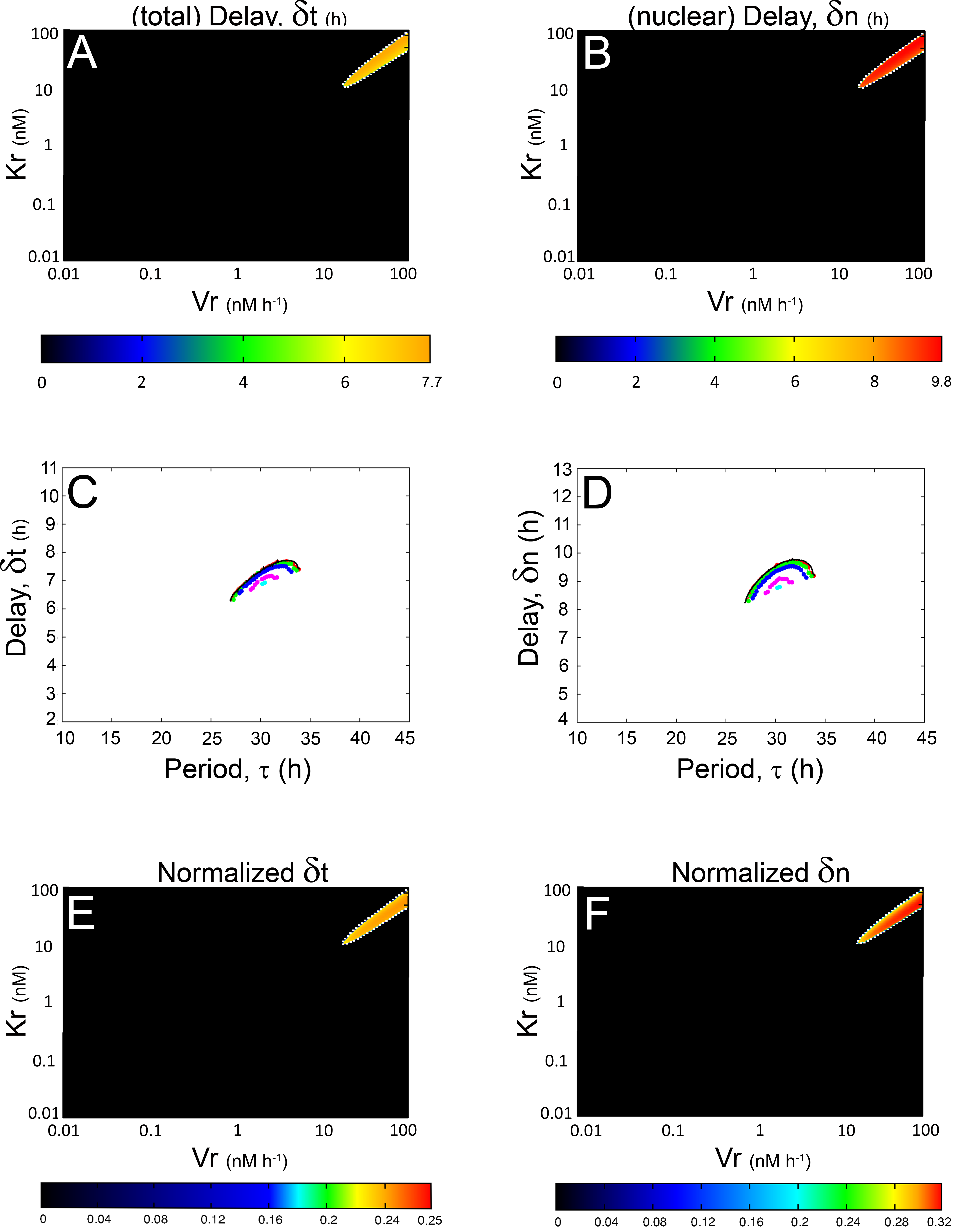
**

**Figure S9**

**
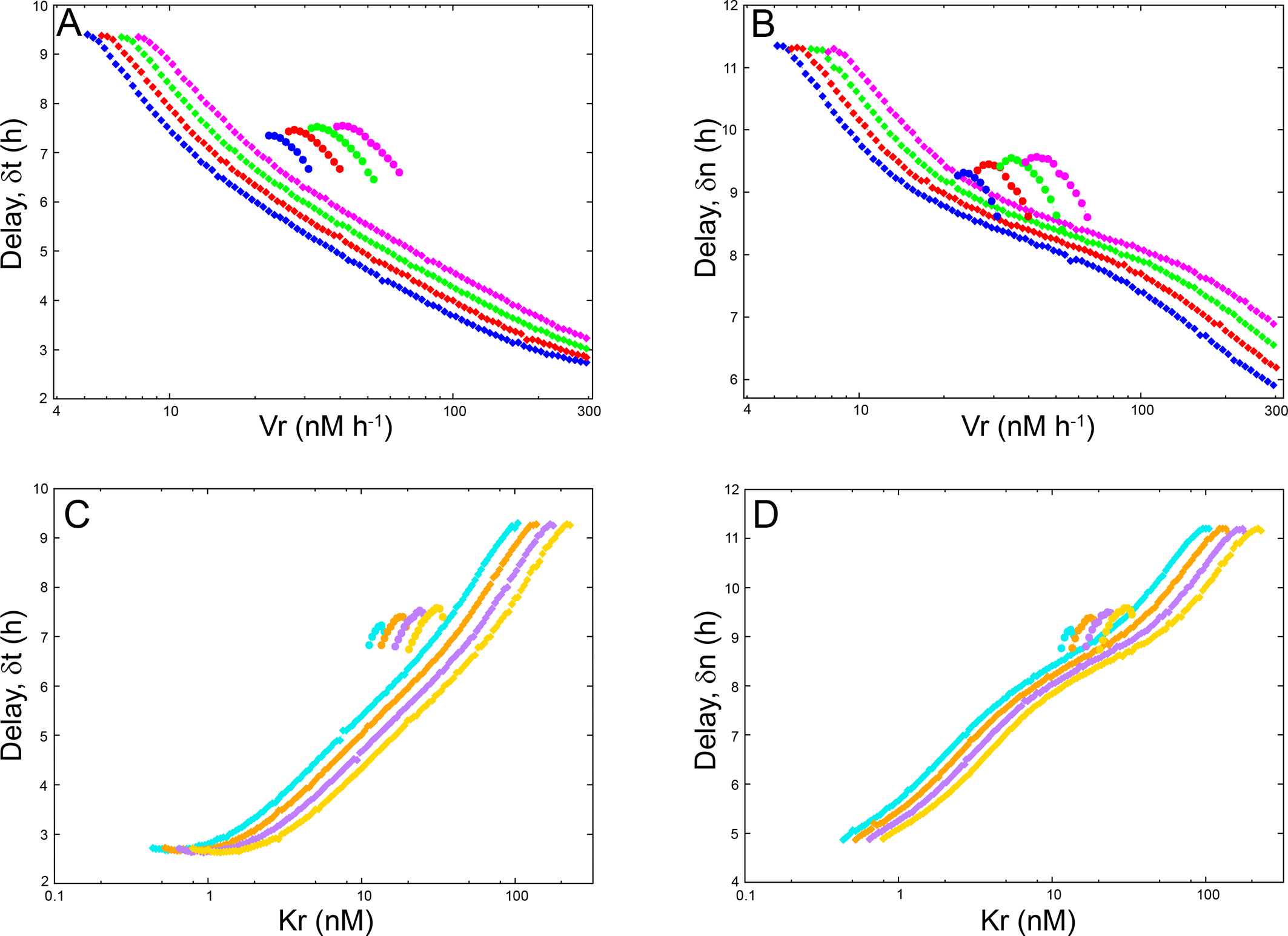
**

**Figure S10**

**
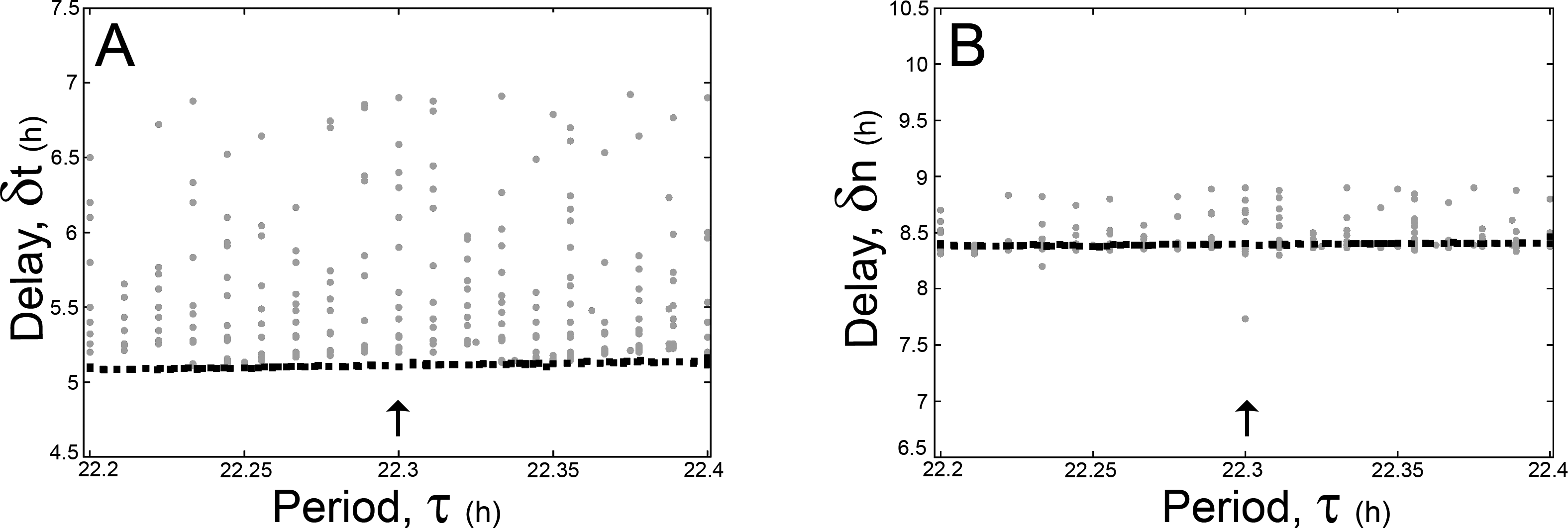
**

**Figure S11**

**
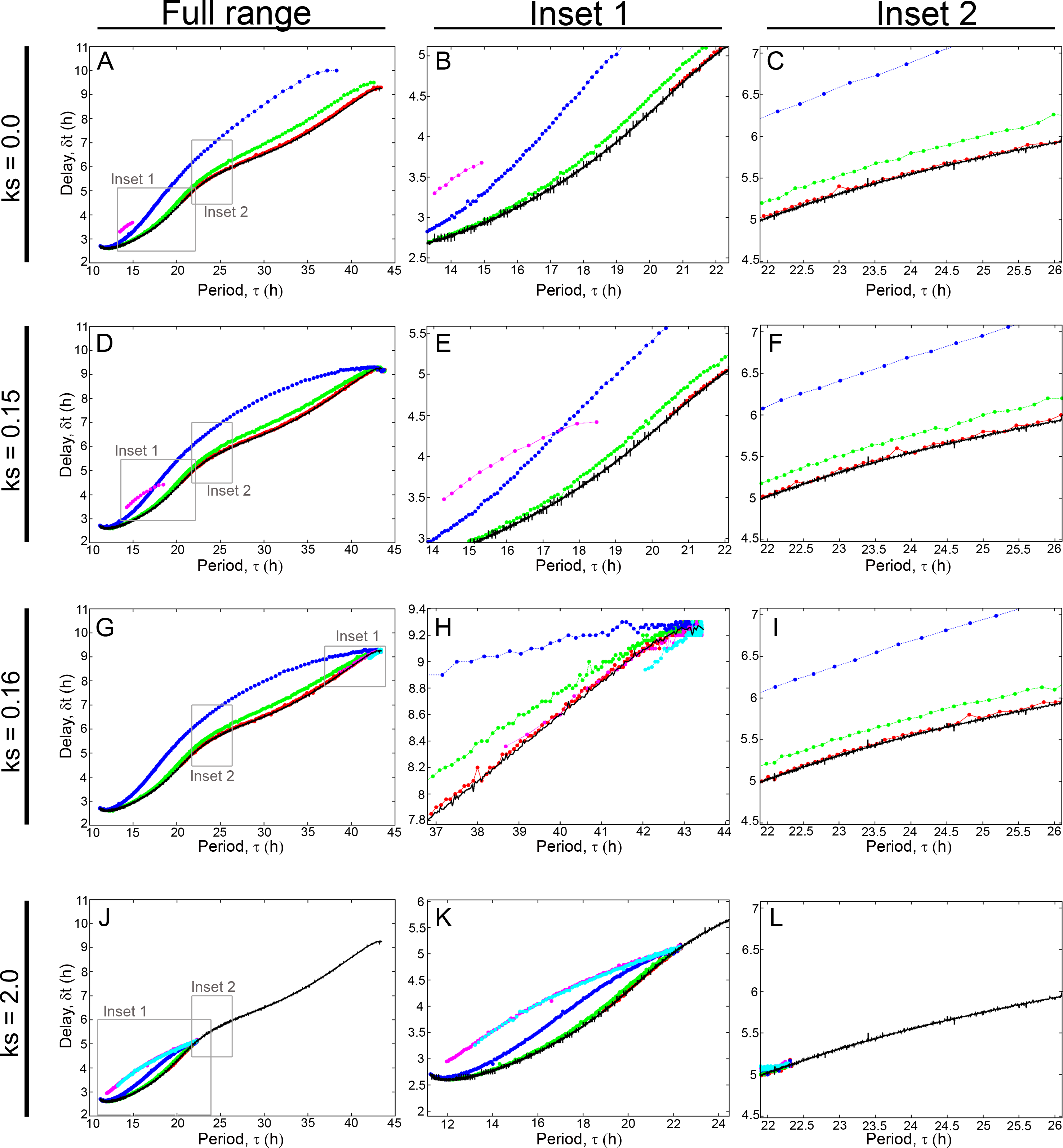
**

**Figure S12**

**
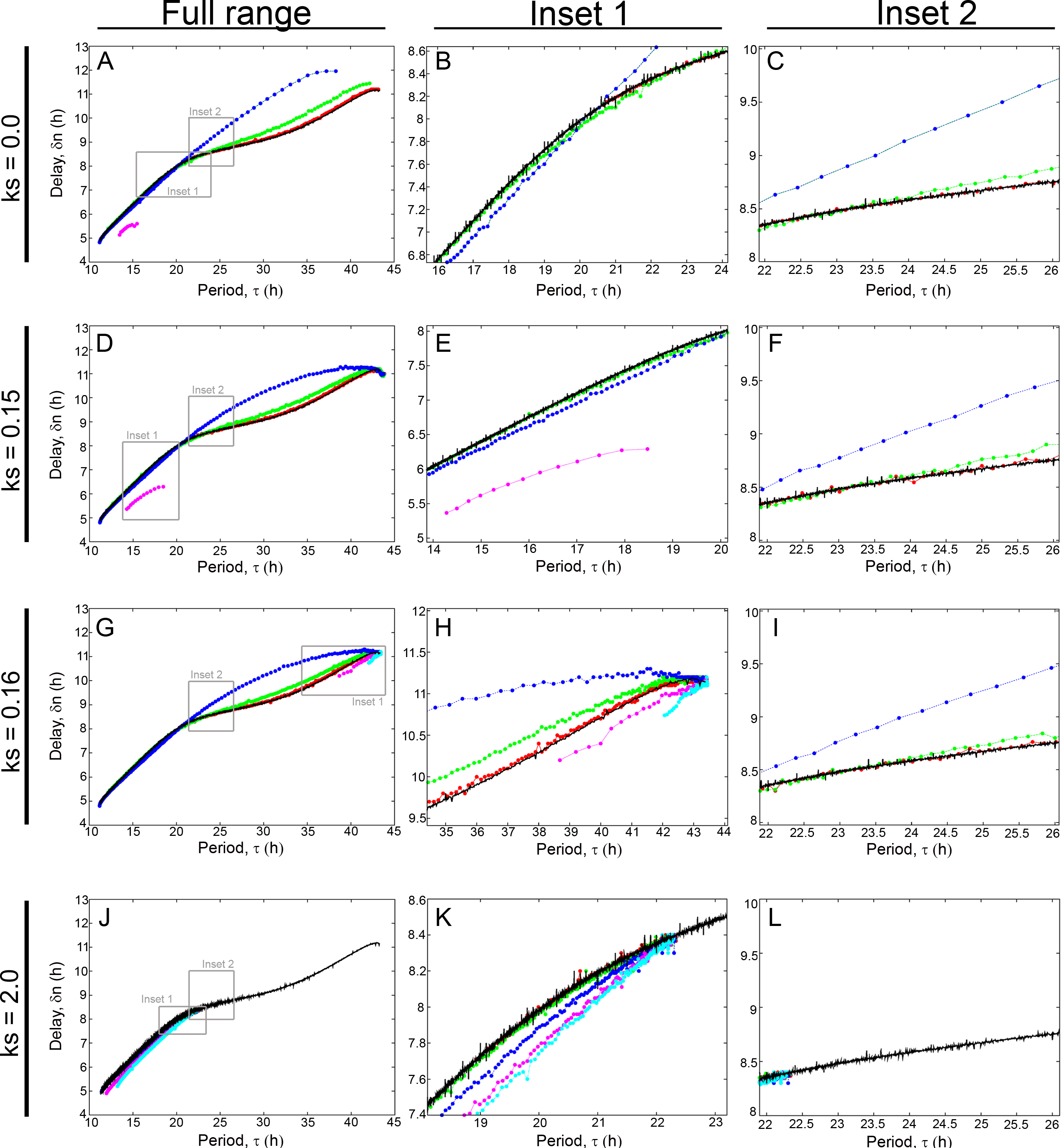
**

**Figure S13**

**
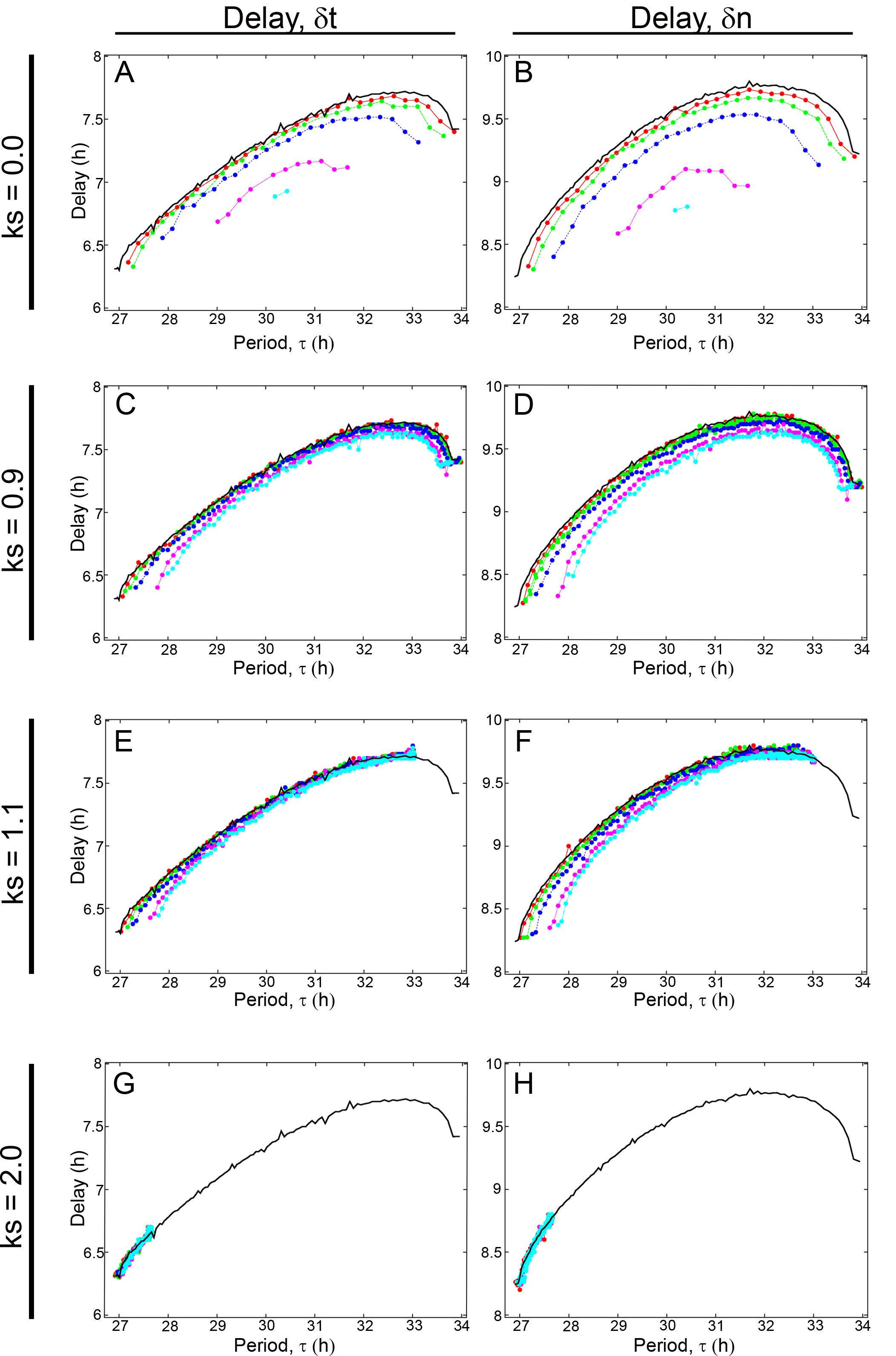
**

**Figure S14**

**
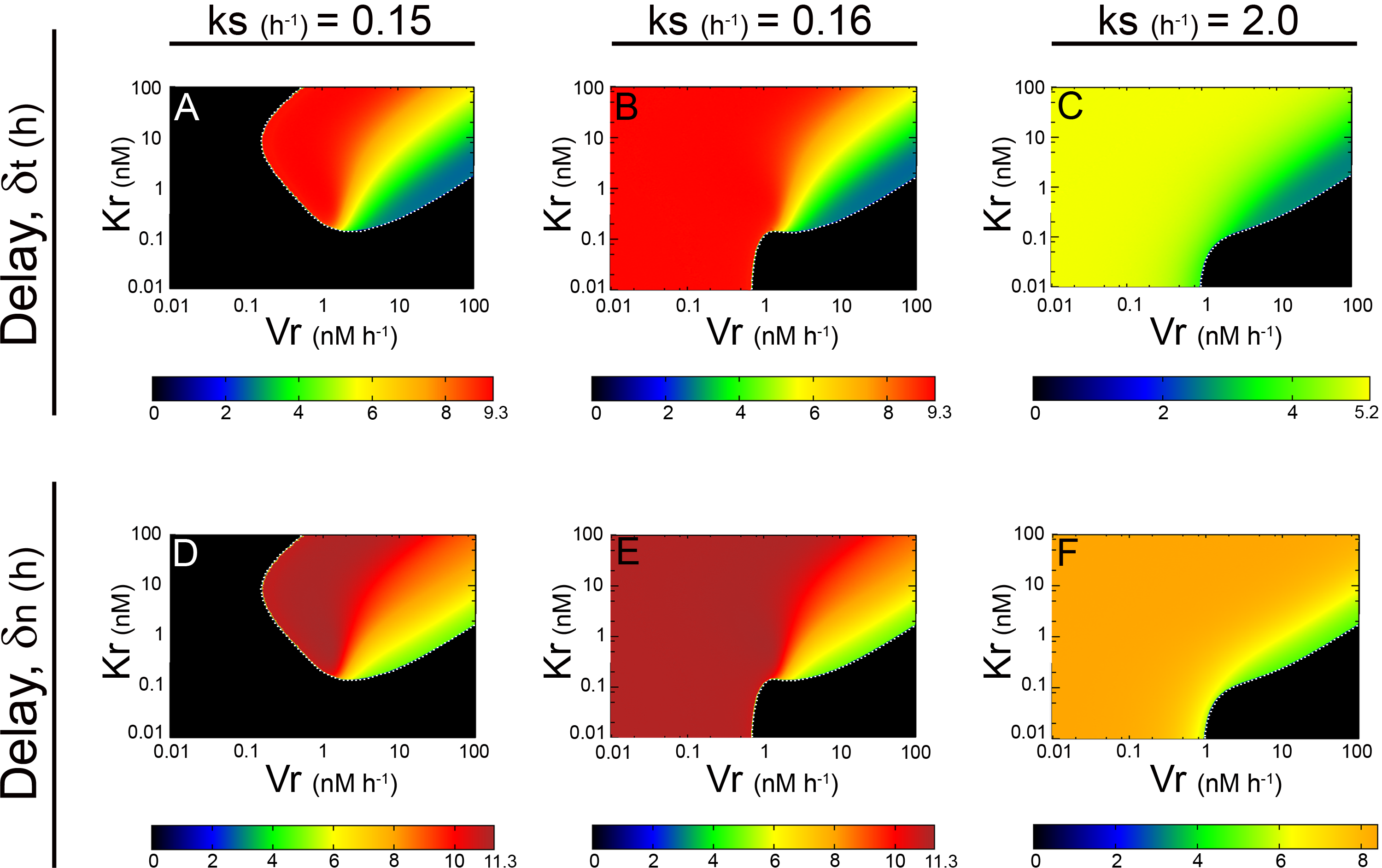
**

**Figure S15**

**
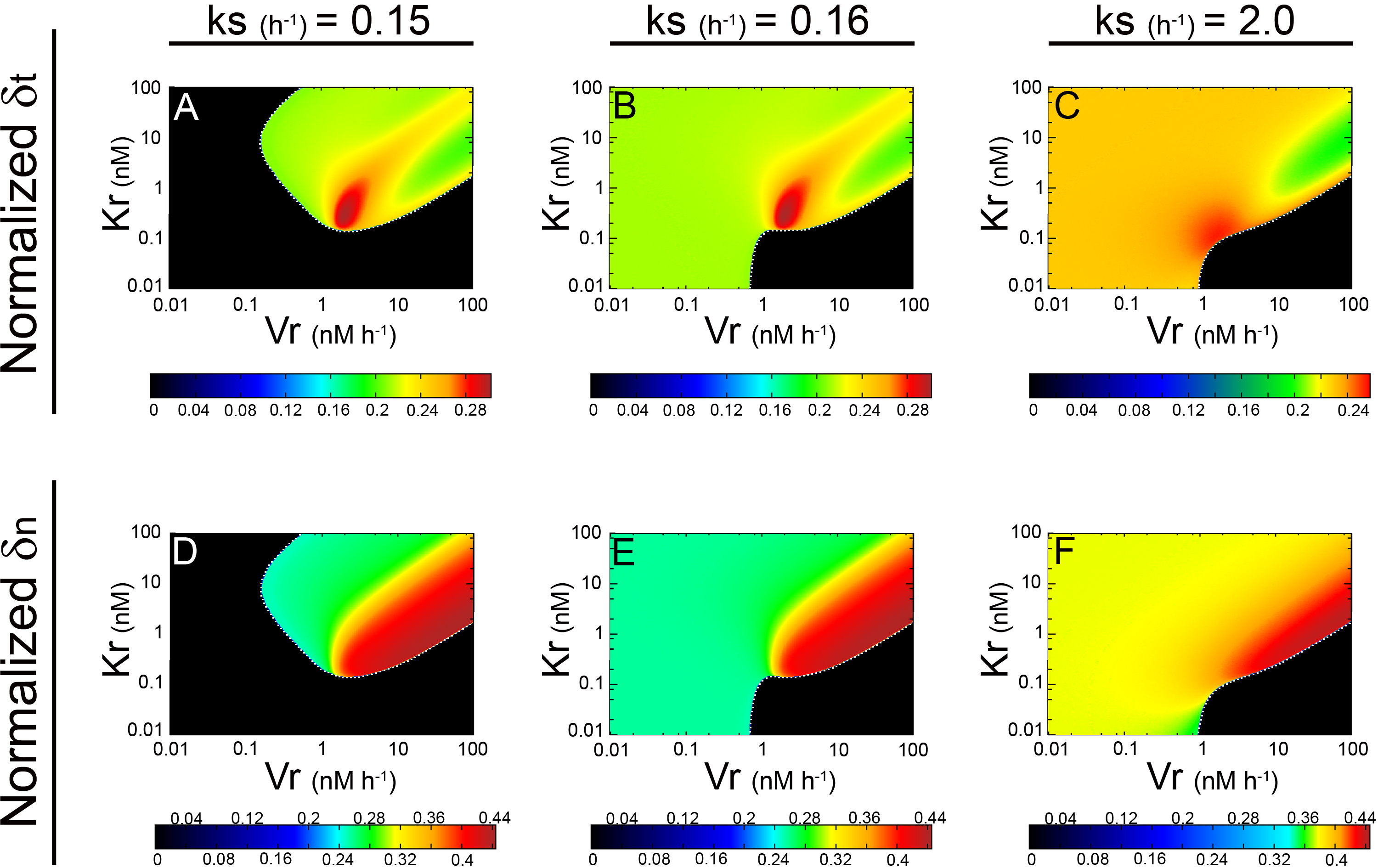
**

**Figure S16**

**
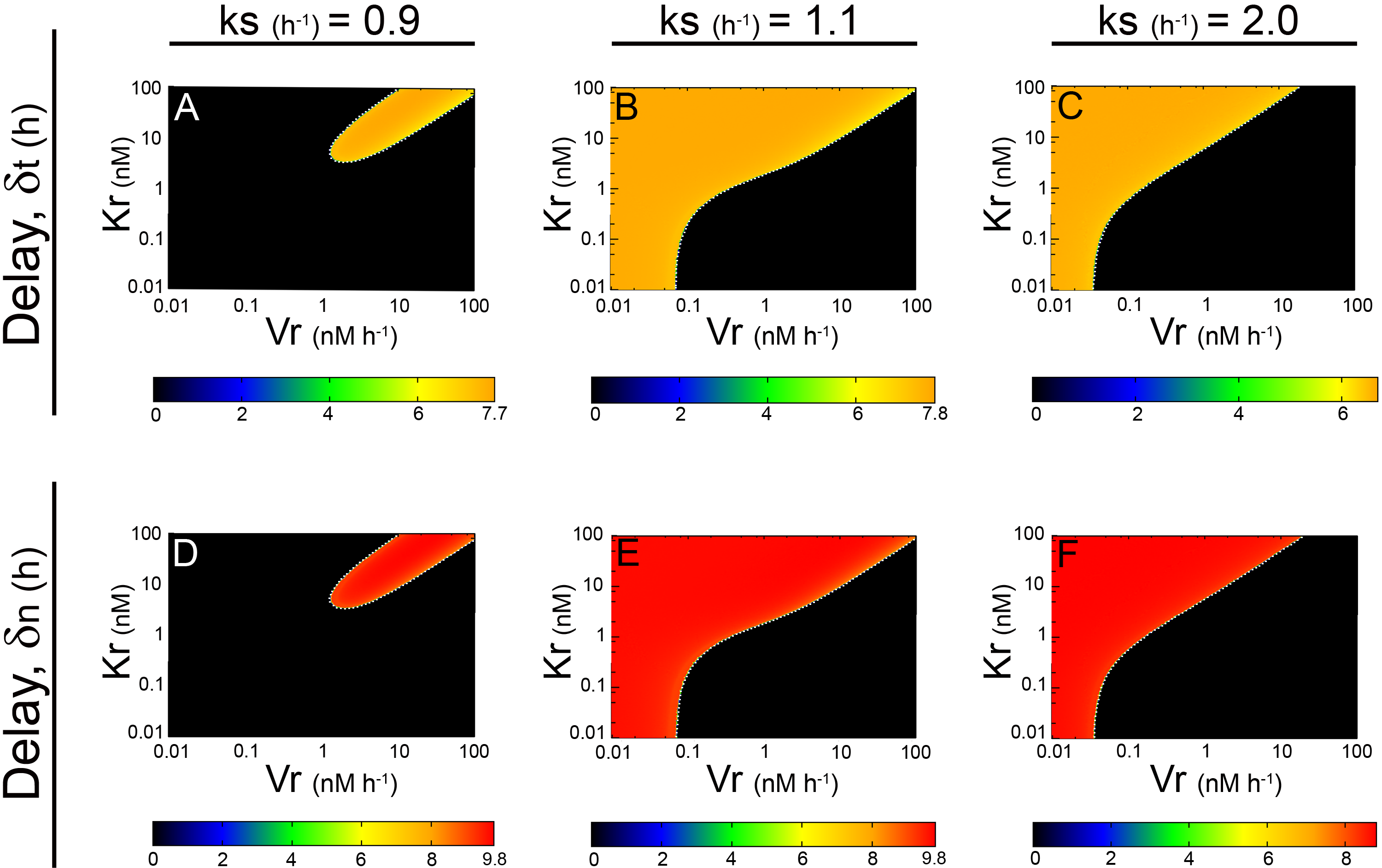
**

**Figure S17**

**
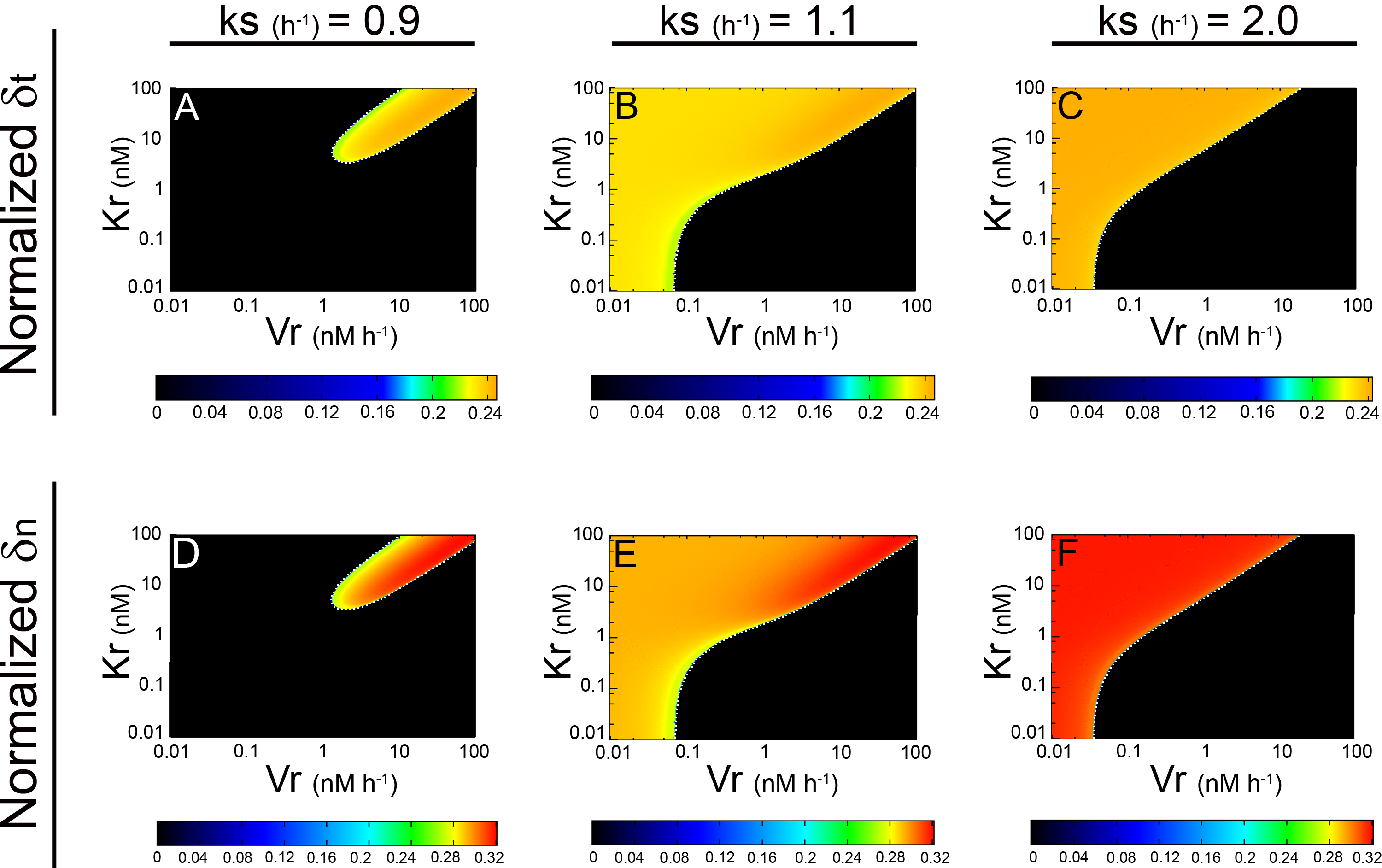
**

**Figure S18**

**
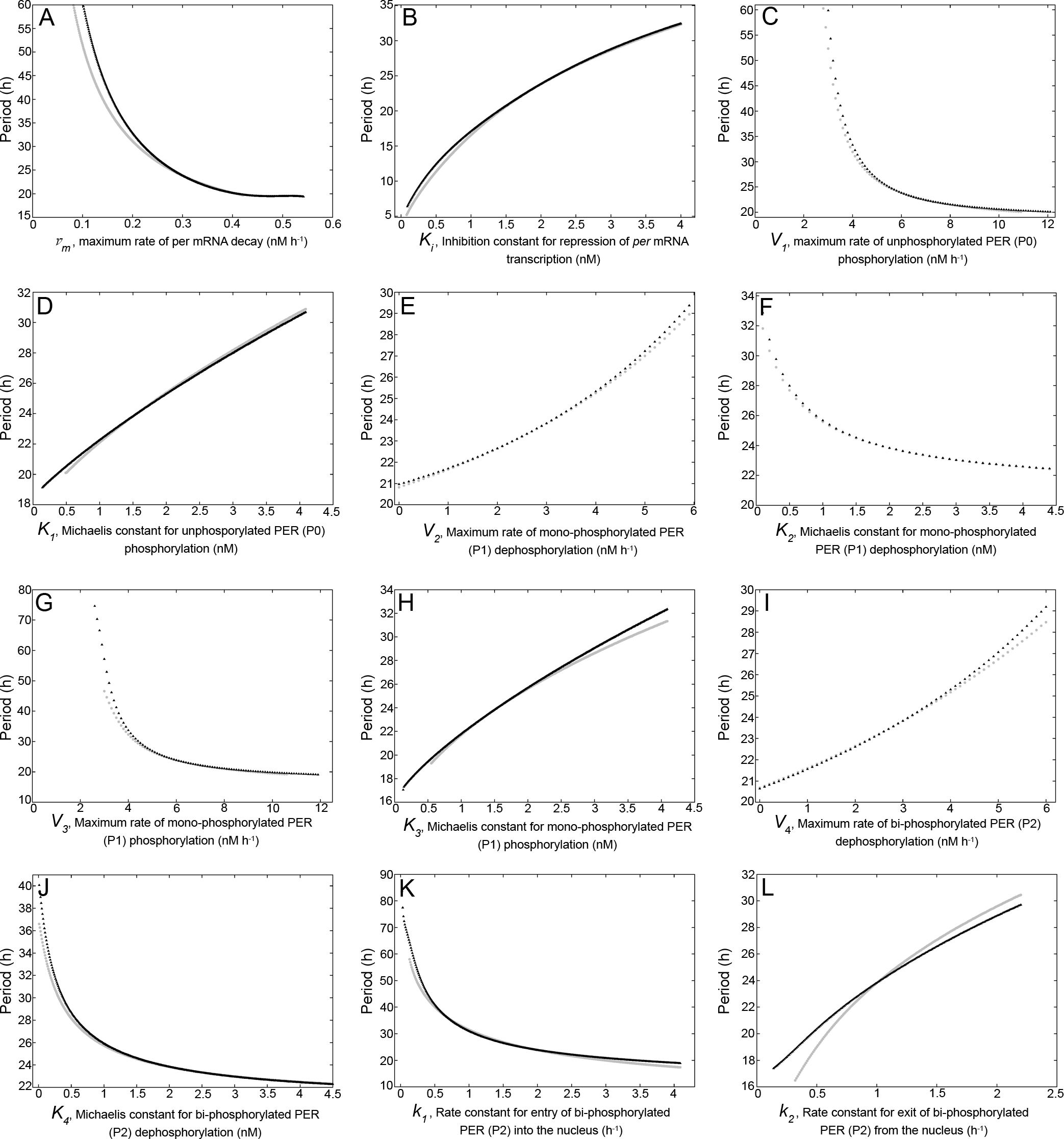
**
